# Lipid-Derived Mediators in Endogenous Anti-Inflammation and Resolution: Lipoxins and Aspirin-Triggered 15-epi-Lipoxins

**DOI:** 10.1100/tsw.2002.81

**Published:** 2002-01-22

**Authors:** Charles N. Serhan, Nan Chiang

**Affiliations:** Center for Experimental Therapeutics and Reperfusion Injury, Department of Anesthesiology, Perioperative and Pain Medicine, Brigham and Women’s Hospital, Harvard Medical School, Boston, Massachusetts 02115, USA

**Keywords:** lipid mediators, leukocytes, lipoxins

## Abstract

It is well appreciated that lipid-derived mediators play key roles in inflammation and many other physiologic responses where multicellular processes are involved. Among them, lipoxins (LX) and aspirin-triggered LX (ATL) evoke actions of interest in a range of physiologic and pathophysiologic processes, and these two series have emerged as founding members of the first class of lipid/chemical mediators “switched on” in the resolution phase of an inflammatory reaction. These unique compounds possess a trihydroxytetraene structure and are both structurally and functionally distinct among the many groups of lipid-derived bioactive mediators. LXA4 and 15-epi-LXA_4_ (a member of the ATL series) display leukocyte-selective actions that enable them to serve as endogenous “stop signals” in multicellular events in that they modulate adherence, transmigration, and chemotaxis. Both LXA_4_ and 15-epi-LXA_4_ elicit these responses via a G protein-coupled receptor (GPCR), termed ALXR, identified in human and murine tissues. Among eicosanoids, ALXR is stereoselective for LXA_4_ (5S,6R,15S-trihydroxy-7,9,13-trans-11-cis-eicosatetraenoic acid). Its aspirin-triggered 15R epimer (15-epi-LXA_4_) and their bioactive stable analogs act in the subnanomolar to nanomolar range in human cellular systems and murine models of acute inflammation and reperfusion. ALXR also has the ability to interact with a wide panel of small peptides that give different signaling responses in vitro than LXA_4_ or its analogs, suggesting that ALXR is capable of serving as a multirecognition receptor in immune responses. Characterization of ALXR and development of metabolically stable LX and ATL analogs that are mimetics rapidly advanced our appreciation of the mechanism of LX actions and the potential utility of these counter-regulatory biocircuits in the quest to control local inflammatory events. In this on-line update, LX and ATL biosynthesis and the LXA_4_ specific receptor, termed ALXR, are reviewed with a focus on their roles in inflammation and resolution with respect to pharmacology, molecular biology, and signal transduction in several cell types and animal models investigated thus far.

[†]For additional information and updates, see: http://letheon.bwh.harvard.edu/research/overview/cet+ri.phtml and http://serhan.bwh.harvard.edu/

### INTRODUCTION*

Inflammation initially involves the recognition of self and nonself by leukocytes. It is now clear that a diverse range of endogenous chemical mediators control these events and orchestrate the host response[1]. These small chemical signals regulate leukocyte traffic as well as the cardinal signs of inflammation. It is well established that the classic eicosanoids such as prostaglandins (PG) and leukotrienes (LT) play important roles and exert a wide range of actions in responses of interest in inflammation[2]. In recent years, the scope and range of chemical mediators identified has expanded considerably[1] to include novel lipid mediators, many new cytokines and chemokines, gases (i.e., nitric oxide and carbon monoxide), and reactive oxygen species as well as new roles for nucleotides as mediators such as adenosine[3,4,5] and the most recently uncovered of this class, namely inosine monophosphate (IMP), that also regulates neutrophil (PMN) trafficking[6] (see Illustration 1 and ***http://serhan.bwh.harvard.edu***). Many of these chemical signals are held to be proinflammatory when generated in elevated levels, as in disease. Interactions between each of these classes *in vivo* remain for the most part largely unexplored and are likely to engage and produce many new levels of control as well as potential novel signals (Illustration 2). Along these lines, Krump et al.[5] found that endogenous adenosine is a very potent inhibitor of LT formation by PMN. Also, IMP inhibited cytokine-initiated PMN infiltration and attenuated PMN rolling in microvessels[6].

A body of results from the author's laboratory[7,8] and other investigators demonstrated that endogenous mediators are generated to dampen the host response and orchestrate resolution[1,9,10]. In this regard, the lipoxins (LX) were the first to be identified and recognized as endogenous anti-inflammatory lipid mediators relevant in resolution in that they can function as "braking signals" or chalones in inflammation[7]. Most recent evidence with clinical and experimental exudates revealed early coordinate appearance of LT and PG with PMN recruitment. This was followed by LX biosynthesis, which was concurrent with spontaneous resolution. Human peripheral blood PMN exposed to $PGE_2$ (as in exudates) switched eicosanoid biosynthesis from predominantly $LTB_4$ and 5-lipoxygenase(LO)-initiated pathways to $LXA_4$, a 15-LO product that "stopped" PMN infiltration (Illustrations 3 and 5). These results indicate that functionally distinct lipid mediator profiles switch during acute exudate formation to "reprogram" the exudate PMN to promote resolution.

It is of particular interest that aspirin (ASA), a widely used nonsteroidal anti-inflammatory drug (NSAID) with many beneficial properties[11] in addition to its well-appreciated ability to inhibit PG[12], also triggers the endogenous generation of 15-epimeric LX, termed ASA-triggered LX (ATL)(Illustration 5, 6, and 7). This occurs via acetylation of cyclooxygenase-2 (COX-2) at sites of inflammation *in vivo*[13] (*vide infra*) that carry anti-inflammatory and antiproliferative actions[14,15]. This is a previously unappreciated and novel mechanism of drug action that has intriguing implications for targeted drug design. But more importantly, they help to further illustrate the importance of endogenous generation of lipid mediators with anti-inflammatory properties. The traditional approach to developing anti-inflammatory drugs, as in other human conditions amenable to pharmacologic interventions, is the use of biosynthesis inhibitors and receptor antagonists of proinflammatory mediators, which indeed have enjoyed

*__*ABBREVIATIONS:*__* ALXR, lipoxin $A_4$ receptor; ASA, aspirin; ATL, aspirin-triggered 15-epi-lipoxins, BLT, leukotriene $B_4$ receptor; COX, cyclooxygenase; GPCR, G protein-coupled receptor; LC/MS/MS, liquid chromatography-tandem mass spectrometry; LM, lipid mediators; LO, lipoxygenase; LT, leukotriene; LX, lipoxin; MPO, myeloperoxidase; PAF, platelet-activating factor; PMN, neutrophils

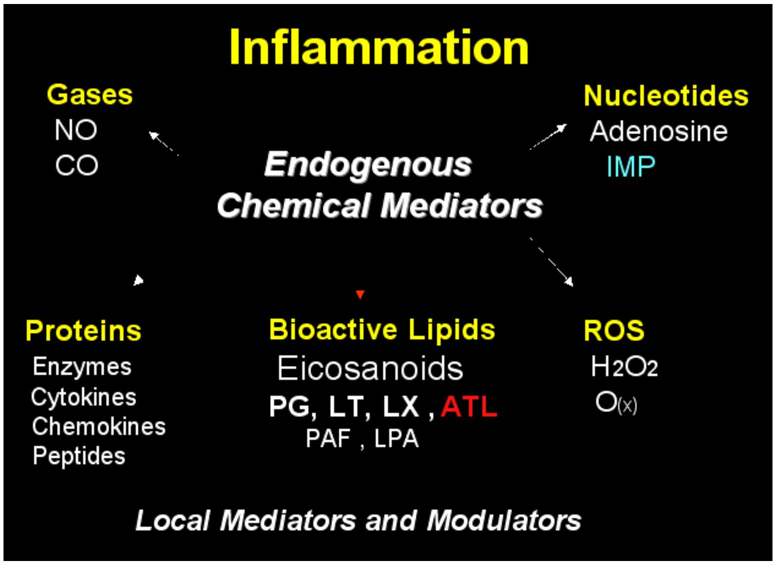

**Illustration 1.*** The classes of compounds now held to play important roles as mediators or signaling compounds in inflammation have increased in recent years to include gases and nucleotides as well as the members of the well established classes of lipid mediators, proteins (wide range of chemokines and cytokines), and reactive oxygen species (ROS).

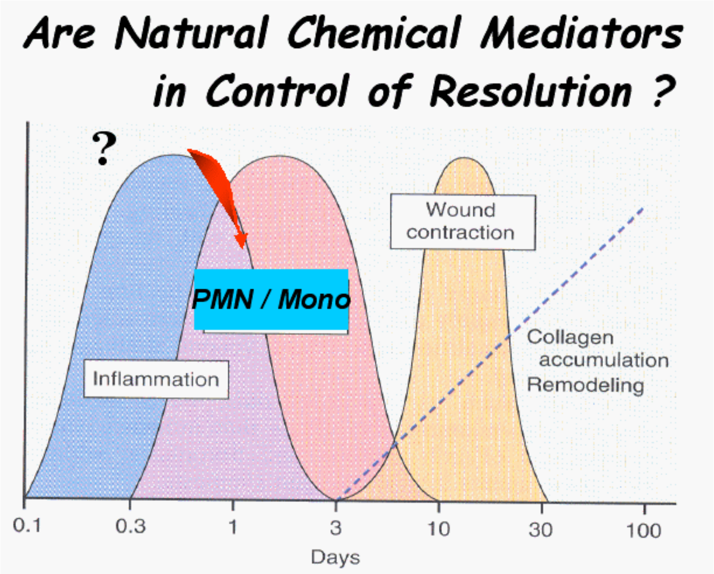

**Illustration 2.**

both considerable clinical and commercial successes[1,16], but are not without significant unwanted side effects[17,18,19]. Hence, the emergence of endogenous pathways and cellular mechanisms involved in counterregulation of responses that can lead to tissue injury and acute inflammation not only charts relatively unappreciated sides of human biology[20,21], but also provides an opportunity to explore new therapeutic approaches based on these novel endogenous mechanisms that may reduce the possibilities for unwanted toxic side effects and help control inflammation with a high degree of precision.

* **Note to reader:** These illustrations/figures were prepared to run in parallel to the text with seven tables to help facilitate the use of this material. For the animated version of these illustrations, please go to our Web site, http://serhan.bwh.harvard.edu.

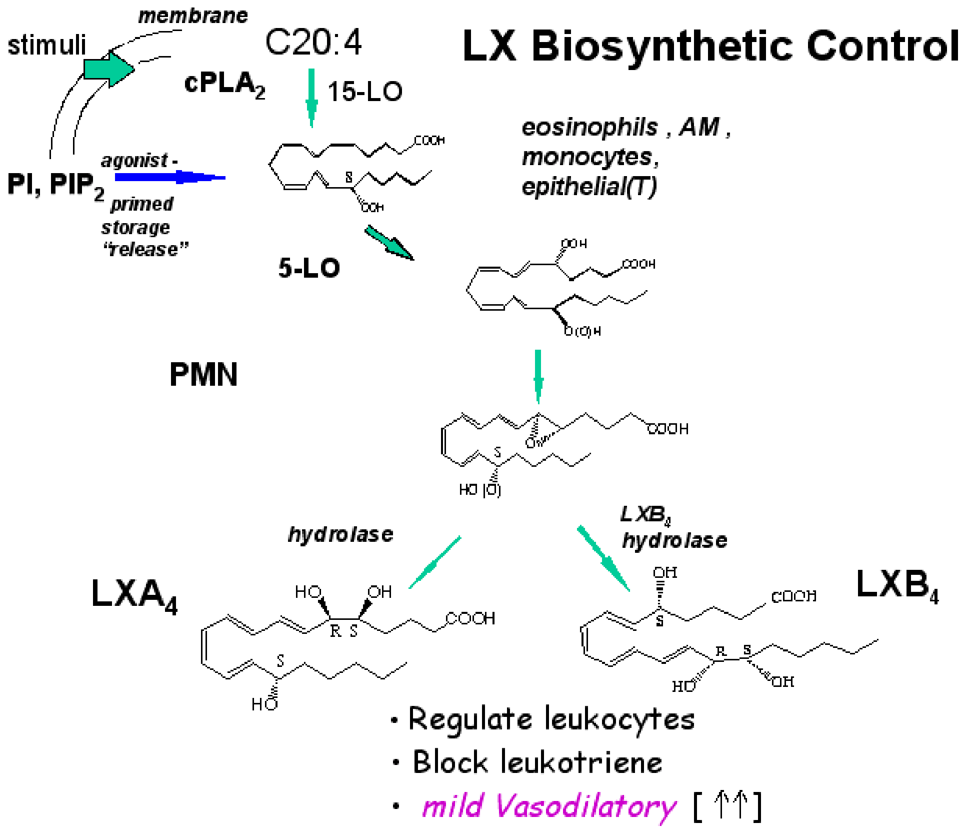

**Illustration 3.** $LXA_4$ and a positional isomer, $LXB_4$, are generated during transcellular biosynthesis initiated by 15-LO. This event also blocks LT formation and therefore regulates leukocytes.

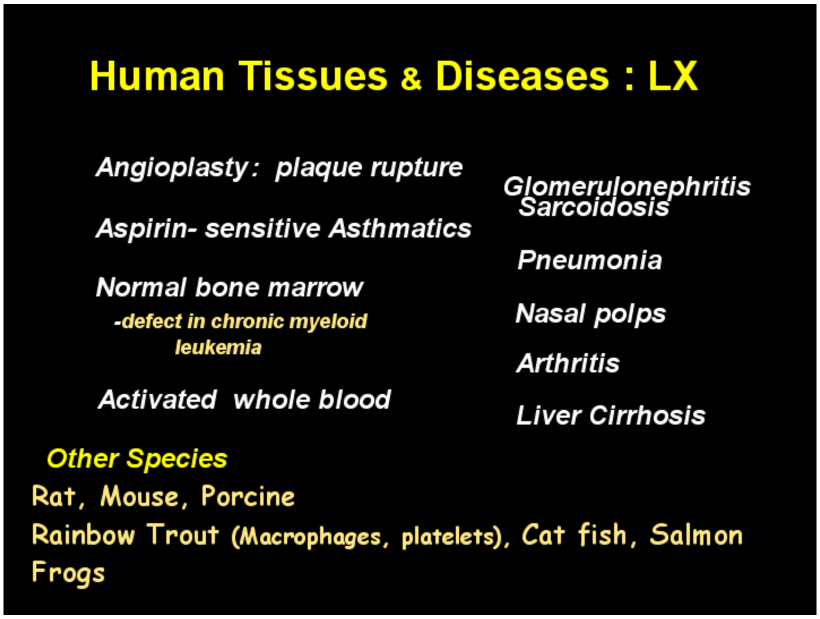

**Illustration 4.** Alterations in LX levels are associated to the pathophysiology of several human diseases. The LX structure is conserved in evolution and is produced by several other species.

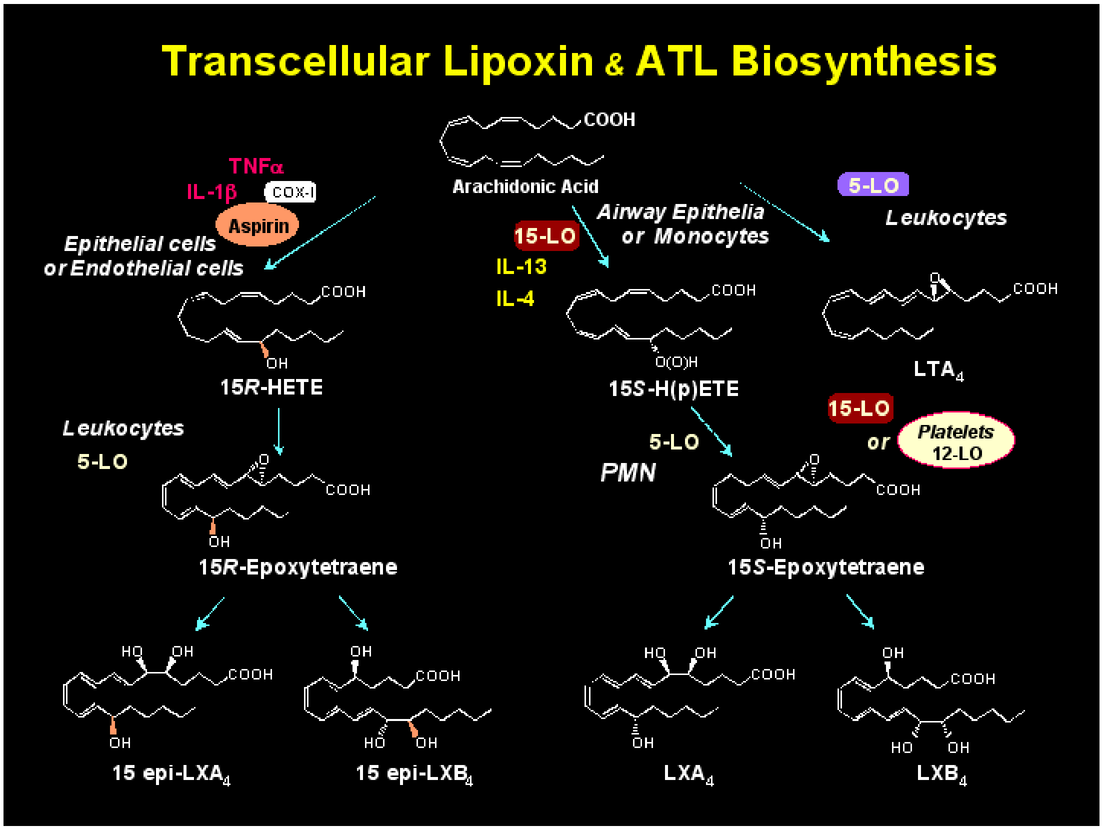

**Illustration 5.** Two main pathways (right and middle) appear to be used in human cells and tissues to generate LXs. 15-epi-LXs are generated when COX-2 is upregulated after acetylation by ASA (left).

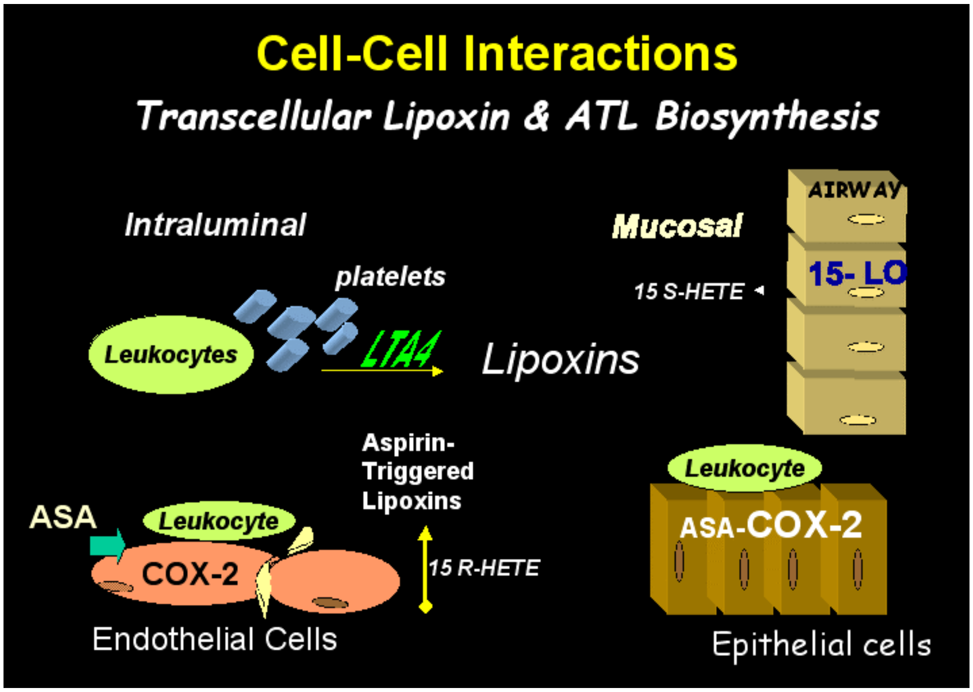

**Illustration 6.** During cell-cell interactions, LX and their endogenous carbon-15 position epimers ATL can be amplified by transcellular biosynthesis via the interactions of two or more cell types.

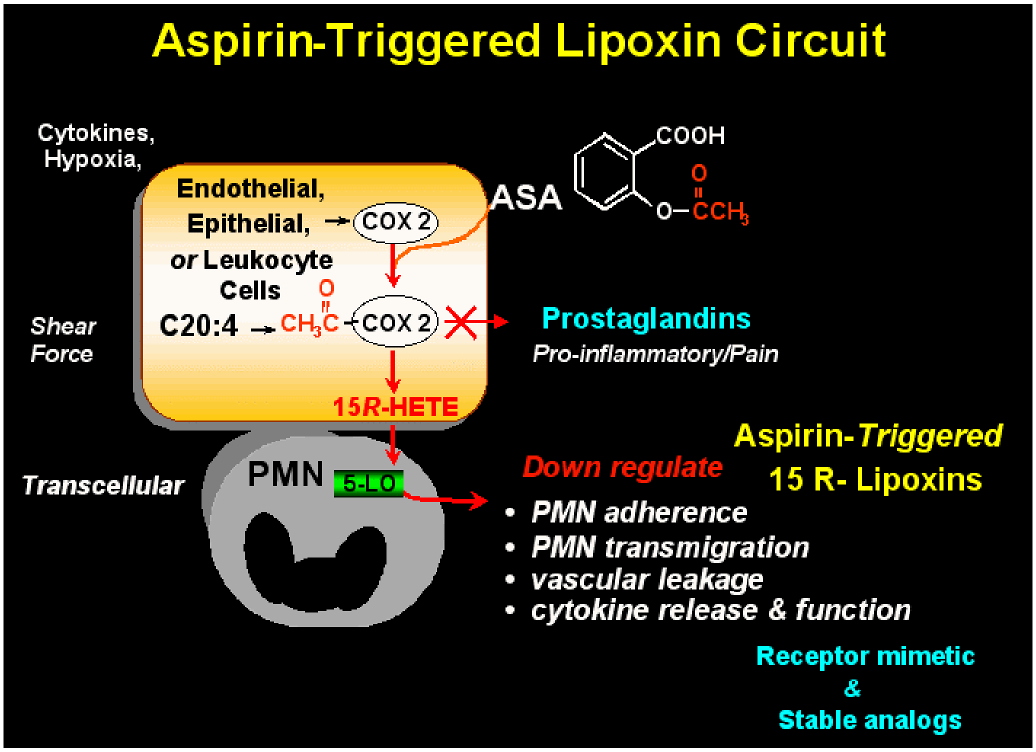

**Illustration 7.** Irreversible acetylation of COX-2 by ASA changes the enzyme's product from PG intermediate to precursors of ATL. The acetylated COX-2 remains catalytically active to generate 15R-HETE (see text).

As a class, LX and their analogs possess physiologic, pathophysiologic, and pharmacological actions in several target tissues. On their discovery, it was not initially clear what the role of these new compounds was since they did not carry bioactions like LT or PG. Each action of LX is stereoselective in that changes in potencies accompany double bond isomerization and change in alcohol chirality (*R* or *S*) at key positions, as well as selective dehydrogenation of alcohols and reduction of double bonds (Illustrations 8, 9, and 10). The self-limited impact of LX in the local microenvironment suggests that they contribute to resolution of injury sites and/or resolve inflammatory loci by regulating further recruitment of PMN and stimulating monocyte migration to promote healing and remodeling. The human PMN and monocyte responses with $LXA_4$ were examined in further detail and will be presented later in our review (*vide infra in silico).*

$LXA_4$ stimulates rapid lipid remodeling within seconds and releases arachidonic acid within PMN but without oxygenation, which is sensitive to pertussis toxin (PTX) treatment[22,23], findings that pointed to the involvement of a G protein-coupled receptor (GPCR) in the actions of LX on human leukocytes. This specific GPCR was identified and cloned in human and mouse, and denoted $LXA_4$ receptor (ALXR). Together, they were identified as the first cloned LO-derived eicosanoid receptors. More recently, using a similar approach as for ALXR enabled identification of the long sought-after human $LTB_4$ receptor (denoted as BLT)[24] and its murine homolog[25].

It is of interest that the BLT displays some sequence homology with ALXR, which was originally cloned as a purinergic receptor[26]. Both ALXR and BLT are more akin to early notions regarding the exclusivity of chemokine receptors and their structures rather than PG receptors (*vide infra;* Illustration 11). Their similarity likely reflects the structures of LT and LX, which contain thermal- and UV-sensitive conjugated double bond systems, whereas PG contain a more chemically stable and less flexible cyclopentane-containing structure. BLT was suggested

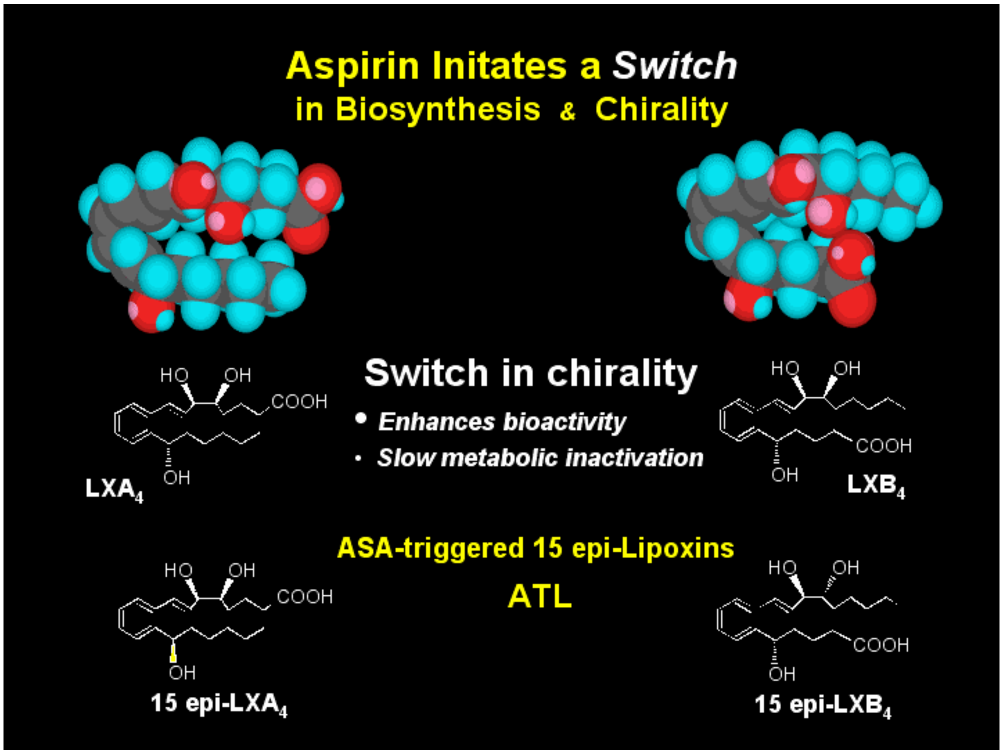

**Illustration 8.** ASA initiates generation of 15-epi-LX that carry their 15 position alcohol in the R configuration, which show enhanced bioactivity and slower metabolic inactivation than 15S native LX. The dehydrogenase is stereospecific and prefers the S rather than R 15 epimer while the ALXR is more accommodating in handling both epimers.

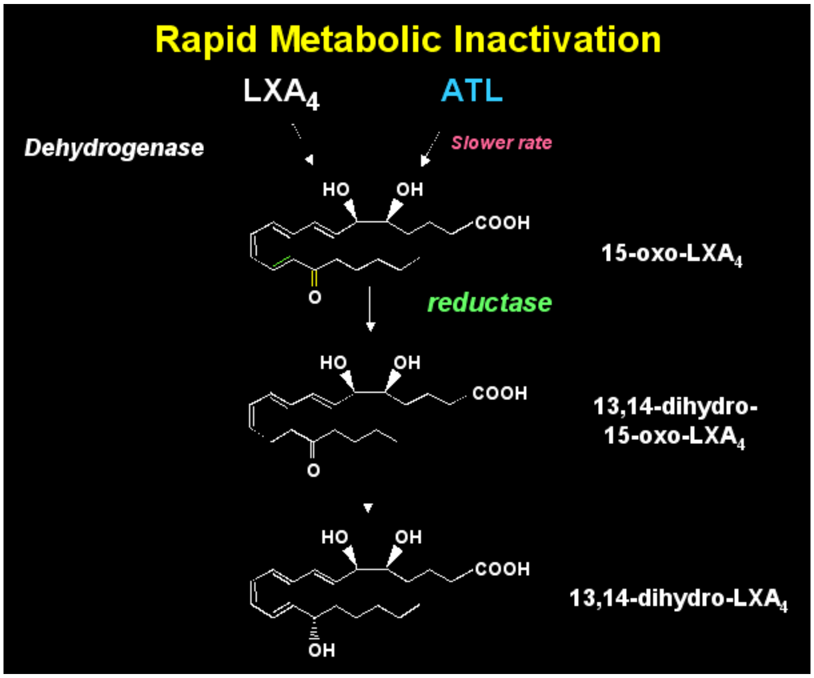

**Illustration 9.** The initial step in $LXA_4$ inactivation is dehydrogenation of the 15-hydroxyl group, catalyzed by 15-PGDH to yield 15-oxo-$LXA_4$. PGR/$LTB_4$DH catalyzes the reduction of the 13,14 double bond of 15-oxo-$LXA_4$ to give 13,14-dihydro-15-oxo-$LXA_4$. This product serves as a substrate to 15-PGDH, which catalyzes the reduction of the C15 oxo-group to give 13,14-dihydro-$LXA_4$. These metabolites appear to be inactive as inhibitors of PMN.

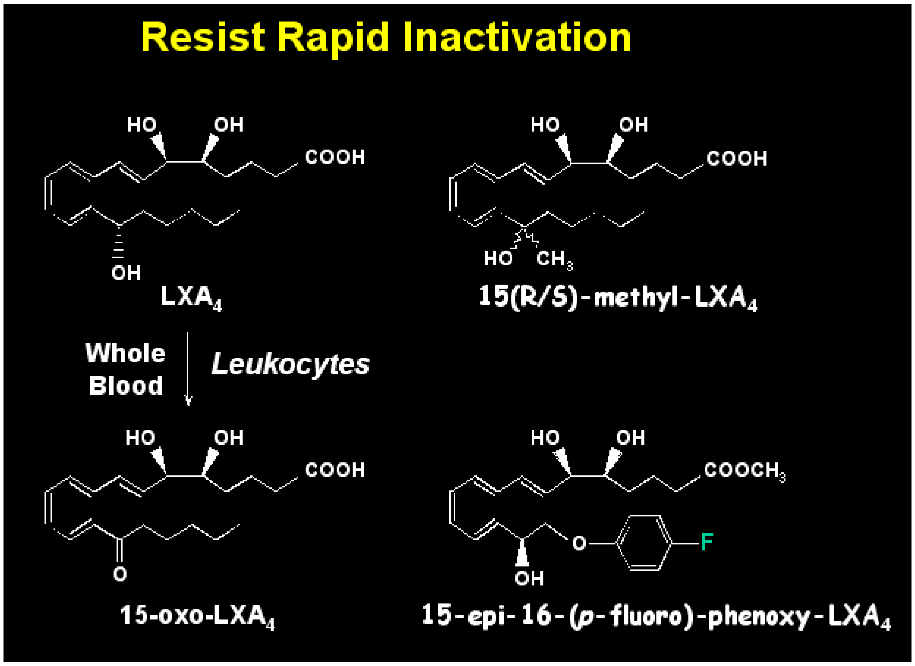

**Illustration 10.** Metabolic stable analogs of both $LXA_4$ and 15-epi-$LXA_4$ (shown on the right) were designed to resist rapid inactivation at carbon-15 and the ω-end of the molecule[20,53]. For $LXB_4$ analogs that resist rapid inactivation, see Serhan et al.[20] and Maddox et al.[52]

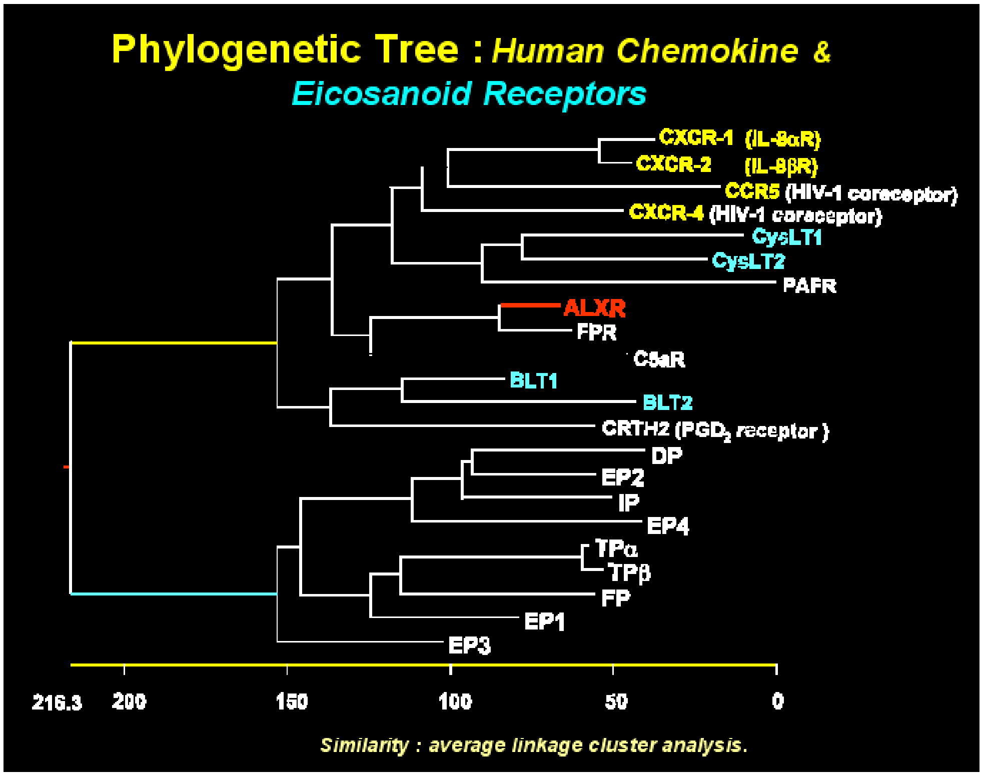

**Illustration 11.** Structural similarity of deduced amino acid sequences of human eicosanoid and chemokine receptors is determined by the average linkage cluster analysis. Abbreviations: TP (thromboxane $A_2$ receptor), $EP_1$, $EP_2$, $EP_3$, $EP_4$ (subtypes of $PGE_2$ receptor), FP ($PGF_2$ receptor), IP (prostacyclin receptor), ALXR (lipoxin $A_4$ receptor), and BLT ($LTB_4$ receptor). BLT, CXCR-4, and CCR-5 were identified as coreceptors for HIV-1 entry.

as a novel coreceptor mediating HIV-1 entry into CD4-positive cells[27]. Along these lines, ALXR was also recently shown to interact with specific peptide and protein ligands[28]. These observations suggest that receptors of this class can interact with both small endogenous lipophilic ligands of the host as well as larger exogenous protein structures, an interesting aspect of these receptors that might relate to their potential multifunctional roles as sensing receptors in host defense. Here, we provide an update and overview of current knowledge of the actions of $LXA_4$ and the characterization of one of its seven transmembrane receptors, namely ALXR, that is involved in regulating PMN, monocyte, and epithelial responses.

### BIOSYNTHESIS OF LIPOXINS

#### Transcellular Biosynthesis of LX and 15-epi-LX: The Role of Cell-Cell Interaction

Platelet-leukocyte interactions and/or platelet-leukocyte microaggregates[29] promote the formation of LX by transcellular conversion of the leukocyte (Illustration 5, right side) 5-LO epoxide product $LTA_4$. Once thought to be solely an intracellular intermediate in LT production, it is now clear that $LTA_4$ released by activated leukocytes is available for enzymatic conversion by neighboring cell types[5,30]. When platelets are adherent, their 12-LO converts $LTA_4$ to $LXA_4$ and $LXB_4$. For a review and mechanistic details with recombinant 12-LO, see Serhan[21]. Hence it is important to note that human platelets, which do not produce LX on their own, become a major source of LX, given their abundance *in vivo* and their highly active 12-LO.

15-LO-initiated LX production is illustrated (Illustration 5, middle right) by airway epithelial cells, monocytes, or eosinophils, which upregulate their 15-LO when exposed to cytokines such as IL-4 or IL-13[31,32]. The 15-LO, by definition, inserts molecular oxygen at the carbon-15 position of, for example, arachidonic acid, in the "*S*" configuration. When these cell types are activated, they generate and release 15*S*-HETE[7,14], which is rapidly taken up and converted by PMN to LX via the action of their 5-LO. This event not only leads to LX biosynthesis, but also "turns off" LT formation. 5-LO conversion of 15*R*-HETE also results in inhibition of LT biosynthesis[15]. 15*R*-HETE is a major product of arachidonic acid in several cell types when COX-2 is upregulated after acetylation by ASA (Illustration 5, left side). Thus, it is possible that ASA can regulate the *in vivo* production of LT by 15*R*-HETE conversion to 15-epi-LX, and 15-epi-LX can in turn also regulate the cellular actions of LT.

We sought evidence for alternate explanations for ASA therapeutic actions because many beneficial new actions have been documented in recent clinical studies. These new potential therapeutic indicators for ASA include decreasing incidence of lung, colon, and breast cancer (reviewed by Levy[33]), and prevention of cardiovascular diseases[34]. Inhibition of COX and biosynthesis of PG can account for many of ASA therapeutic properties[35]; however, its ability to regulate PMN-mediated inflammation or cell proliferation remains of interest. Along these lines, we uncovered a new action of ASA that involves COX-2-bearing cells such as vascular endothelial cells or epithelial cells and their coactivation with PMN (Illustration 6 and 7). Hence, inflammatory stimuli (i.e., TNF$\alpha$, LPS, etc.) induce COX-2 to generate 15*R*-HETE when ASA is administered[14]. This intermediate carries a carbon-15 alcohol in the *R* configuration that is rapidly converted by activated PMN to 15-epi-LX, or LX that carry their 15 position alcohol in the *R* configuration[21] rather than 15*S* native LX, which in humans can result from LO:LO interaction, the molecular oxygen inserted mainly in the *S* configuration.

$LXB_4$ is a positional isomer of $LXA_4$, carrying alcohol groups at carbon 5*S*, 14*R*, and 15*S* positions, instead of the C-5*S*, 6*R*, and 15*S* positions present in $LXA_4$. ASA-triggered $LXB_4$ carries a 15*R* alcohol, hence 15-epi-$LXB_4$ (Illustrations 5 and 8). Although $LXA_4$ and $LXB_4$ show similar activities in some biologic systems[36], in many others each shows distinct actions([37]

and reviewed in [21]). 15-epi-$LXB_4$, for example, is a more potent inhibitor of cell proliferation than $LXA_4$ or 15-epi-$LXA_4$[21]. Next in this review we shall focus on findings indicating that 15-epi-$LXA_4$ is generated in inflammatory exudates in an ASA-dependent manner and that ASA-triggered $LXA_4$ and novel fluorinated $LXA_4$ as well as $LXB_4$ stable analogues are potent, topically active inhibitors of PMN-directed actions *in vivo*.

#### Aspirin (ASA)-Dependent Generation of 15-epi-$LXA_4$ *In Vivo*

To determine whether 15-epi-$LXA_4$ could be detected in animal experimental models, experiments were first carried out with a mouse peritonitis model[13]. In this model of inflammation, COX-2 protein levels were upregulated by intraperitoneal injection of lipopoly-saccharide (LPS). Peritonitis was induced by intraperitoneal injection of casein. To test whether ASA treatment of the mice results in the generation of 15-epi-$LXA_4$ during an inflammatory event, ASA was administrated by intraperitoneal injection. The collected peritoneal exudates from each mouse were incubated in the presence or absence of the agonist ionophore A23187 without addition of exogenous substrates, and samples from individual mice were analyzed separately using a newly developed specific ELISA method and LC-MS-MS system[13].

Given one or two doses of ASA[13], the mean values for 15-epi-$LXA_4$ production were ~1.5 and 1.8 ng/5 ml peritoneal lavage per mouse. Without administration of ASA, approximately 0.5 ng of 15-epi-$LXA_4$ per 5 ml lavage was associated with peritoneal exudates from each mouse, suggesting that additional routes may be operative *in vivo* to produce 15-epi-LX in an ASA–independent fashion. Naive animals (without any treatment) gave very low levels (<0.2 ng/5 ml peritoneal lavage per mouse) of 15-epi-$LXA_4$. The physiological relevance of these values obtained in the absence of experimental challenge is currently not clear. These results demonstrated that ASA administration in murine peritonitis gives inflammatory exudates that generate 15-epi-$LXA_4$ in appreciable levels from endogenous substrate within these inflammatory cells, thus establishing a biosynthetic circuit for ATL/15-epi-LX generation *in vivo*.

### LX AND HUMAN DISEASES

These methods (e.g., LC/MS/MS and ELISA) were recently used to evaluate ATL and $LXA_4$ formation in ASA-tolerant and -intolerant asthmatics and their relation to $LTC_4$. Of interest, the ASA-tolerant subjects generated both LX and ATL, but the ASA-intolerant patients proved to have a diminished capacity to generate ATL and LX upon ASA challenge[38]. The lower levels of these potentially protective mediators could contribute to the pathobiology of this chronic disorder in that the disease state is not only characterized by the overproduction of proinflammatory mediators but the loss or reduction in LX and ATL that may keep inflammation in check. Also, a reduction and alteration in LX generation was found in patients with chronic liver disease[39] and chronic myelogenous leukemia[40,41,42,43,44]. These diseases contrast with recent findings that $LXA_4$ production is up-regulated in localized juvenile periodontitis[45, see Illustration 18] as well as following atherosclerotic plaque rupture[46], and with nasal polyps[47]. Together, this result indicates that alterations in LX levels may be linked to the pathophysiology of several human diseases and may display local organ-specific functions that stand apart from their roles in inflammation and within local inflammatory lesions (Table 1). In this context, the ability of both $LXA_4$ and $LXB_4$ and their stable analogs to lower intraocular pressure may underlie their role in the physiology of ocular pressure regulation within the eye[48,49]. In human eye tissues, the receptor ALXR is indeed present and appears to be associated with corneal epithelial cells[50]. LX are evolutionarily conserved in several species of fish and frogs (see Illustration 4 and review in [7,21]), findings that again raise the possibility that the function of LX in humans is fundamental; yet, our knowledge of their range of actions in humans is still evolving.

**TABLE 1**
**Lipoxins and Human Diseases**

| Organ/System | Impact in vivo | Reference |
|---|---|---|
| **Hematologic and Oncologic** | Defect in LX production with cells from chronic myeloid leukemia patients in blast crisis | Stenke et al., 1991 [41] |
| | LX stimulate nuclear form of PKC in erythroleukemia cells | Beckman et al., 1992 [98] |
| | Formation of LX by granulocytes from eosinophilic donors | Serhan et al., 1987 [99] |
| **Vascular** | Angioplasty-induced plaque rupture triggers LX formation | Brezinski et al., 1992 [46] |
| **Renal** | LX trigger renal hemodynamic changes generated in experimental glomerular nephritis | Katoh et al., 1992 [100] |
| | Increased LX excretion in rat kidney transfected with rh15-LO | Munger et al., 1999 [101] |
| **Dermatologic** | $LXA_4$ regulates delayed hypersensitive reactions in skin | Feng et al., 1996 [102] |
| | LX inhibit PMN infiltration and vascular permeability | Takano et al., 1997 [55] |
| **Pulmonary** | $LXA_4$ detected in bronchoalveolar lavage fluids from patients with pulmonary disease and asthma | Chavis et al., 1995 [103] |
| | Production of LX by nasal polyps and bronchial tissue | Edenius et al., 1990 [47] |
| | $LXA_4$ inhalation shifts and reduces $LTC_4$-induced contraction is asthmatic patients | Christie et al., 1992 [104] |
| | Aspirin-intolerant asthmatics display a lower biosynthetic capacity than aspirin-tolerant patients | Sanak et al., 2000 [38] |
| **Hepatic** | LX generation decreased in cirrhotic patients | Claria et al., 1998 [39] |
| **Rheumatoid arthritis** | LX levels increase with recovery | Thomas et al., 1995 [105] |
| **Ocular** | $LXA_4$ reduces intraocular pressure | Cotran et al., 1995 [48, 49] |
| **Localized juvenile periodontitis** | $LXA_4$ production is upregulated | Pouliot et al., 2000 [45] |

## DESIGN OF STABLE ANALOGS OF $LXA_4$ AND 15-EPI-$LXA_4$: LX AND ATL ANALOGS

### Mechanism of $LXA_4$ Inactivation

LX, as other autacoids, are rapidly biosynthesized in response to stimuli, act locally, and then are rapidly enzymatically inactivated. The major route of $LXA_4$ inactivation is through dehydrogenation by monocytes that convert $LXA_4$ to 15-oxo-$LXA_4$, followed by specific reduction of the double bond adjacent to the ketone[20] (Illustrations 9 and 10). 15-Hydroxy/oxo-eicosanoid oxidoreductase (15-PGDH) catalyzes the oxidation of $LXA_4$ to 15-oxo-$LXA_4$. This compound is biologically inactive and is further converted to 13,14-dihydro-15-oxo-$LXA_4$ by the action of $LXA_4$/PGE 13,14-reductase/$LTB_4$ 12-hydroxydehydrogenase (PGR/$LTB_4$DH). Moreover, reduction of the 15-oxo-group by 15-PGDH yields 13,14-dihydro-$LXA_4$, revealing an additional catalytic activity for this enzyme[51]. $LXB_4$ can also be dehydrogenated by 15-PGDH at carbon-5 to produce 5-oxo-$LXB_4$, therefore sharing a common route of inactivation[52]. It has recently been shown that 15-oxo-$LXA_4$ is also produced from $LXA_4$ in mouse whole blood[53] suggesting that the mouse shares with the human a common pathway for $LXA_4$ inactivation (Illustration 10).

## STRUCTURE REQUIREMENTS FOR $LXA_4$ ANTI-INFLAMMATORY ACTIONS

As a class, LX possess physiologic, pathophysiologic, and pharmacological actions in several target tissues. Each action of LX is stereoselective in that changes in potencies accompany double bond isomerization and change in alcohol chirality (R or S) at key positions as well as selective dehydrogenation of alcohols and reduction of double bonds (Table 2). For example, the 15-hydroxyl group is important for anti-inflammatory properties since ASA-triggered $LXA_4$ (15R-$LXA_4$) with the 15-hydroxyl group in the R-configuration as well as 15(R/S)-methyl-$LXA_4$ have been established in several experimental settings to be more potent than native $LXA_4$ (15S-$LXA_4$) *in vitro* and in *vivo*[54,55]. Also, both 15-oxo-$LXA_4$[51] and 15-deoxy-$LXA_4$[20] are biologically inactive in inhibiting superoxide anion generation and transmigration in PMN, respectively. The 13,14-double bond is important since 13,14-dihydro-$LXA_4$ proved to be inactive in inhibiting superoxide anion generation[51]. These pharmacophores for LX anti-inflammatory action (Table 2) are also required for their interaction with ALXR since these biologically inactive isomers (e.g., 15-oxo-$LXA_4$, 15-deoxy-$LXA_4$, and 13,14-dihydro-$LXA_4$) did not bind to ALXR, whereas the active ones (e.g., 15R-$LXA_4$ and 15[R/S]-methyl-$LXA_4$) give specific binding to ALXR, as demonstrated by specific [$^3$H]-$LXA_4$ binding (see Illustration 13).

### Metabolic Stable Aanalogs of $LXA_4$ and 15-epi-$LXA_4$

In view of the rapid transformation and inactivation of the LX by monocytes, and, potentially, other cells *in vivo*, it was highly desirable to design LX analogs that could resist this form of metabolism, maintain their structural integrity, and potentially enhance beneficial bioactions. LX analogs were constructed with specific modifications of the native structures of $LXA_4$ and $LXB_4$, such as the addition of methyl groups on carbon-15 and carbon-5 of $LXA_4$ and $LXB_4$ structures, respectively, to block dehydrogenation by 15-PGDH. For example, 15(R/S)-methyl-$LXA_4$ is a racemic stable analog of both $LXA_4$ and 15-epi-$LXA_4$ (Illustration 10). Additional analogs of $LXA_4$ were synthesized with a phenoxy group bonded to carbon-16 and replacing the ω-end of the molecule. This design permits 16-phenoxy-$LXA_4$ to resist potential ω-oxidation and to be protected from dehydrogenation *in vivo*. Fluoride was added to the para-position of the phenoxy ring to make 16-(para-fluoro)-phenoxy-$LXA_4$ to hinder degradation of the phenoxy ring. The

**TABLE 2**
**Pharmacophores of $LXA_4$'s Anti-Inflammatory Actions**

| *Functional group* | *compound* | *property* |
|---|---|---|
| **15-hydroxyl group** | $LXA_4$<br>15(R)-$LXA_4$<br>15(R/S)-methyl-$LXA_4$<br>15-deoxy-$LXA_4$<br>15-oxo-$LXA_4$ | Inhibit PMN invitro and in vivo<br>More potent than $LXA_4$<br>More potent than $LXA_4$<br>Inactive in inhibiting PMN transmigration<br>Inactive in inhibiting superoxide generation |
| **11,12-cis double bond** | 11-trans-$LXA_4$ | |
| **13, 14-double bond** | 13,14-dihydro-$LXA_4$ | Inactive in inhibiting superoxide generation |

ASA-triggered 15-epi counterpart of 16-(para-fluoro)-phenoxy-$LXA_4$, 15-epi-16-(para-fluoro)-phenoxy-$LXA_4$, was also synthesized (Illustration 10). These modifications not only prolong the half-life of the compounds in blood but also enhance their bioavailabilities as well as bioactivities[53].

The ATL are less effectively converted *in vitro* to their 15-oxo-metabolite than $LXA_4$[20]. This indicates that the dehydrogenation step is highly stereospecific and suggests that, when ATL are generated *in vivo*, their biologic half-life is increased by about twofold greater than that of native $LXA_4$ (Illustration 8), thereby enhancing their ability to evoke bioactions. Hence, biologically stable analogs of LX and ATL can be engineered to enhance their bioactions, which suggests that they are useful tools, and offers leads for developing novel therapeutic modalities. These analogs proved to be active and also to act via competition at ALXR (Illustration 13).

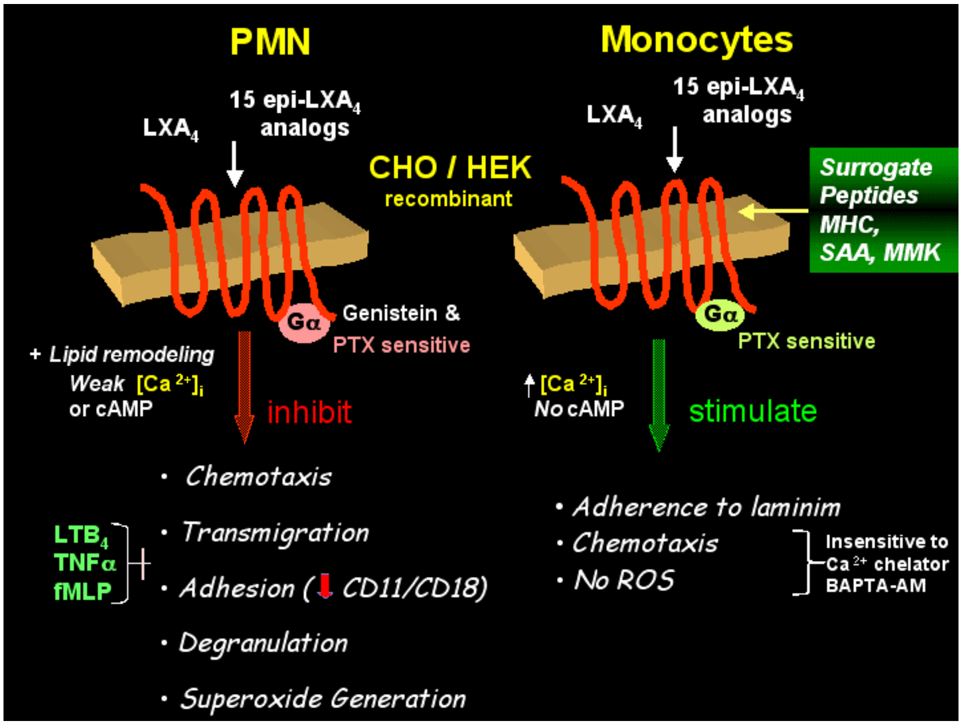

**Illustration 12.** ALXR inhibits PMN and stimulates monocyte functions via PTX-sensitive G proteins (Gα) upon activation by $LXA_4$ and 15-epi-$LXA_4$ as well as LX analogs. In PMN, neither intracellular calcium ($[Ca^{2+}]_i$) nor cAMP was increased in response to LX. In monocytes, $LXA_4$ induced an increase of $[Ca^{2+}]_i$, which is not the second messenger for $LXA_4$-stimulated adherence or chemotaxis since these responses were unaffected by BAPTA-AM (a $Ca^{2+}$ chelator). See text for details.

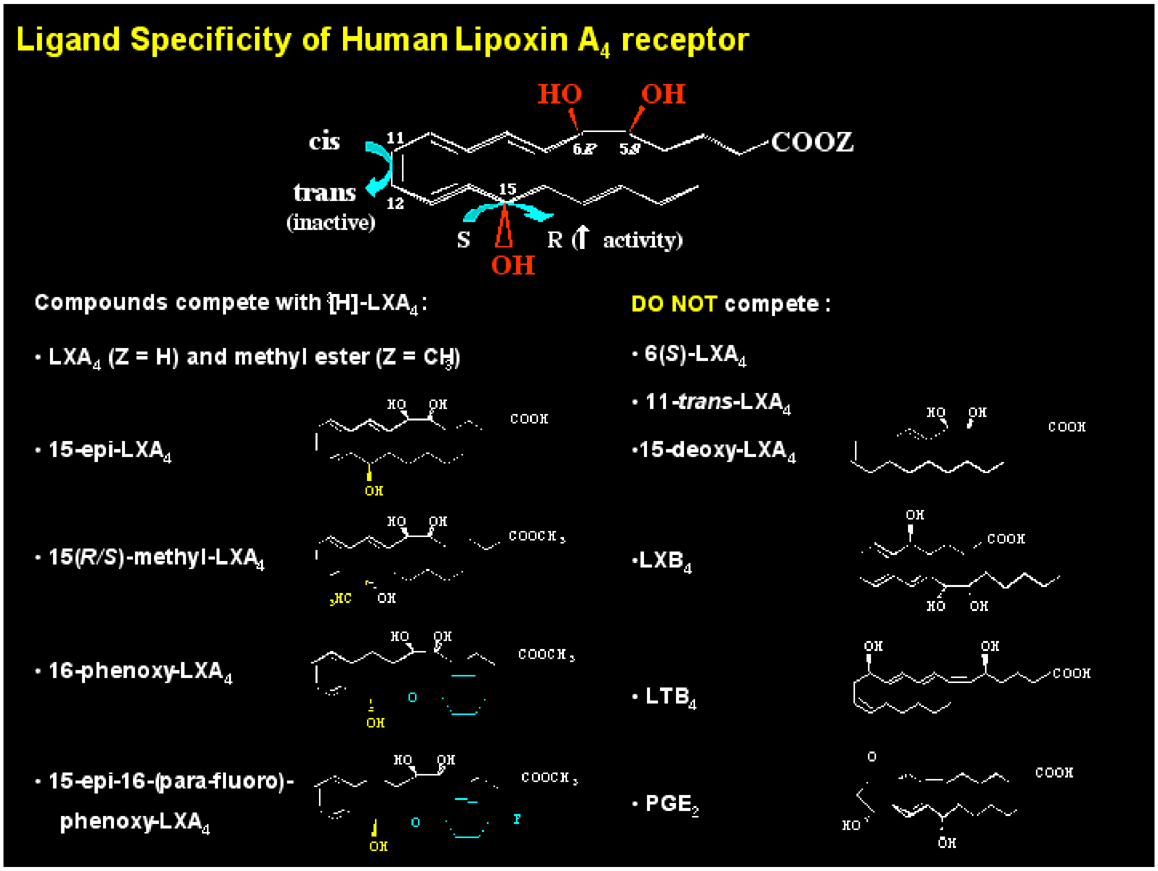

**Illustration 13.** $LXA_4$ interaction with ALXR is highly stereospecific, that is the 5S, 6R-orientation of the two hydroxyl groups as well as 11-cis double bond conformation are essential for bioactions. 15-epi-$LXA_4$ (an ASA-triggered lipoxin, ATL) carries a carbon-15 alcohol at the R configuration, opposite to the S configuration in native $LXA_4$, and was shown to have higher potency than native $LXA_4$ in certain bioassays. In 15(R/S)-methyl-$LXA_4$, hydrogen at carbon -15 was replaced by a methyl group at a racemate at carbon -15. 16-phenoxy-$LXA_4$ has a phenoxyl group at carbon -16. These compounds, which are more resistant to rapid dehydrogenation by 15-hydroxy PG dehydrogenase (15-PGDH) than native $LXA_4$, compete with $[^3H]$-$LXA_4$ specific binding on PMN and are potent inhibitors for PMN functions *in vitro* and *in vivo*.

### LX BIOACTIONS *IN VITRO* AND *IN VIVO*

$LXA_4$ evokes mild vasodilatory and counter-regulatory roles in both *in vivo* (Table 3) and *in vitro* models. These counter-regulatory actions are initiated via unique cell surface receptors on leukocytes and enterocytes (Illustration 14). With other cell types such as endothelium and mesangial cells (Illustration 20), $LXA_4$ evokes bioactions and interacts with a subclass of peptido-LT receptors ($CysLT_1$)[21]. The leukocyte receptors are physiologically and pharmacologically distinct and evoke selective actions on each type of leukocyte tested to date. With human peripheral blood leukocytes, $LXA_4$ inhibits both isolated PMN and eosinophil chemotaxis *in vitro* in the nanomolar range[56,57] and blocks human natural killer (NK) cell cytotoxicity in a stereoselective fashion[21]. In cell-cell interaction systems, $LXA_4$ inhibits PMN transmigration across both endothelial and epithelial monolayers[36,58] via actions on both cell types (i.e., PMN and endothelial cells, PMN and epithelial cells). These responses are also evident *in vivo* with murine receptors[54,55].

When applied topically to mouse ears, these LX stable analogues inhibit both PMN infiltration and vascular permeability changes in a concentration-dependent fashion (Illustration 15)[54,55]. At 130 nmol per ear, the degree of inhibition of PMN infiltration was more than 90%

**TABLE 3**
**Biological Actions of Lipoxins**

| Cell type/Tissue | Action | Reference |
|---|---|---|
| **Neutrophils** | Inhibit chemotaxis, adherence and transmigration | Lee et al., 1989 [56]; Serhan et al., 1995 [20] |
| | Inhibit PMN-epithelial and endothelial cell interactions | Colgan et al., 1993 [58]; Papayianni et al., 1996 [36] |
| | Block superoxide anion generation | Levy et al., 1999 [82] |
| | Inhibit CD11b/CD18 expression and $IP_3$ formation | Fiore & Serhan, 1995 [71]; Grandordy et al., 1990 [22] |
| | Modulate L-selectin expression | Filep et al., 1999 [106] |
| **Monocytes** | Stimulate chemotaxis and adhesion to laminin without increase in cytotoxicity | Maddox et al., 1997 [66] |
| **Eosinophils** | Inhibit migration/chemotaxis | Bandeira-Melo et al., 2000 [107] |
| **NK cells** | Block cytotoxicity | Ramstedt et al., 1987 [108] |
| **Myeloid progenitors** | Stimulate myeloid bone marrow-derived progenitors | Stenke et al., 1994 [44] |
| **Enterocytes** | Inhibit TNF-α-induced IL-8 expression and release | Gronert et al., 1998 [50] |
| | Inhibit Salmonella typhimurium-induced IL-8 | Gewirtz et al., 1998 [79] |
| **Fibroblasts** | Inhibit IL-1β-induced IL-6, IL-8 and MMP-3 production | Sodin-Semrl et al., 2000 [67] |
| **Endothelia (HUVEC)** | Stimulate protein kinase C-dependent prostacyclin formation | Leszczynski & Ustinov, 1990 [109] |
| | Block P-selectin expression | Scalia et al., 1997 [110] |
| **Mesangial cells** | Inhibit $LTD_4$-induced proliferation | McMahon et al., 2000 [78] |
| **Pulmonary artery** | Induce relaxation and reverses pre-contraction by $PGF_2$ or endothelin-1 | Dahlen & Serhan, 1991 [111] |
| **Bronchi** | Relaxation after pre-contraction by peptido-leukotrienes | Christie et al., 1992 [104] |

for both analogues, with apparent $IC_{50}$s noted at ~13 to 26 nmol per ear range for each analogue. In the same concentration range, these two $LXA_4$ stable analogues also inhibited the vascular permeability, namely, extravasation of Evans blue. At 130 nmol per ear, the inhibition of vascular permeability change was >98% for 15(*R/S*)-methyl-$LXA_4$, and ~87% for 16-phenoxy-$LXA_4$, respectively, and their impact was noted visually. The inhibition of vascular permeability changes paralleled inhibition of PMN infiltration with both the ATL and LX analogues. Also, the fluorinated analog of ATL, denoted ATLa, at levels as low as ~24 nmol per mouse, potently inhibited TNF-α-induced leukocyte recruitment into the dorsal air-pouch (Illustration 16)[50]. Inhibition was evident by either local intra-air-pouch delivery (~77% inhibition) or via systemic delivery by intravenous injection (~85% inhibition) and proved more potent than local delivery of ASA. Rank order for inhibiting PMN infiltration was: ATLa (10 μg, i.v.) ≈ ATLa (10 μg, local) ≈ dexamethasone (10 μg, local) > ASA (1.0 mg, local) (Illustration 17). Applied topically to mouse ear skin, ATLa also inhibited PMN infiltration induced by $LTB_4$ (~78% inhibition) or phorbol ester (~49% inhibition), which initiates endogenous chemokine production. Our results indicate that this fluorinated analog of the natural ATL is bioavailable by both local or systemic delivery routes and is a more potent and precise inhibitor of PMN accumulation than ASA *in vivo*[50,54].

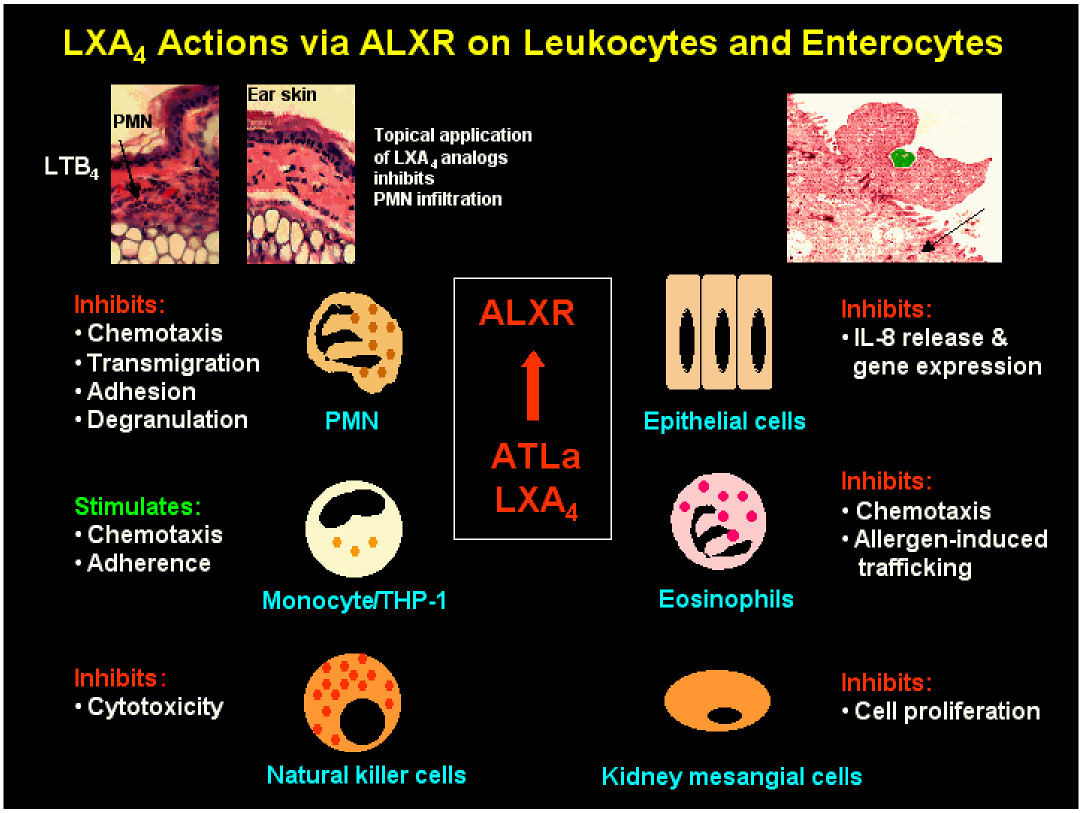

**Illustration 14.** Actions of $LXA_4$ in leukocytes[21] and human epithelial cells[50,79] (upper left panel). Ear biopsies: Inhibition of $LTB_4$-induced PMN infiltration into mouse ear by topical application of $LXA_4$ analogs in acute skin inflammation[55]. PMN is indicated by an arrow (upper right panel). Photomicrograph: Internalization of Salmonella typhimurium (shown in green) by intestinal epithelium (indicated by an arrow). In response to this gastrointestinal pathogen, intestinal epithelium secretes chemokines, which promote PMN infiltration. This chemokine (IL-8) secretion can be downregulated by $LXA_4$ analogs.

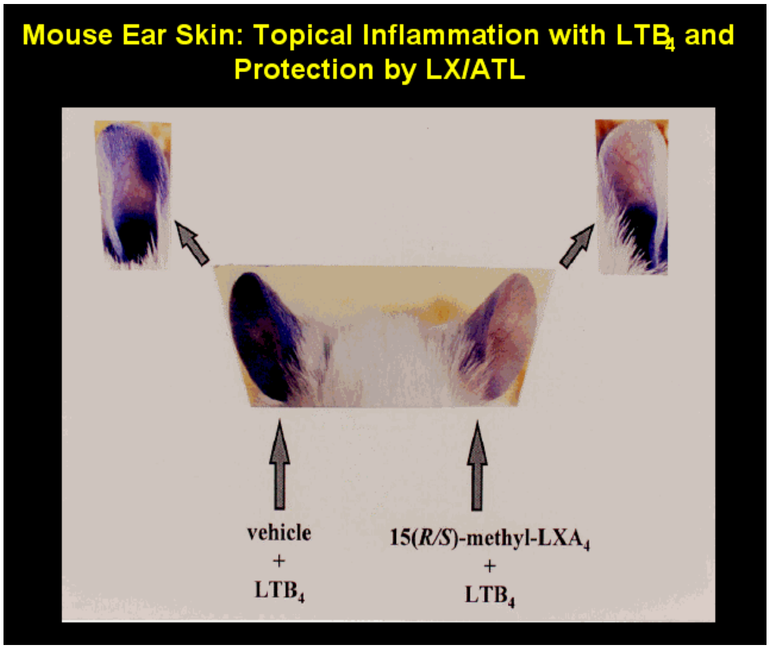

**Illustration 15.** When applied topically to mouse ears, $LXA_4$ stable analogue 15(R/S)-methyl-$LXA_4$ inhibits vascular permeability, visualized by extravasation of Evans blue.

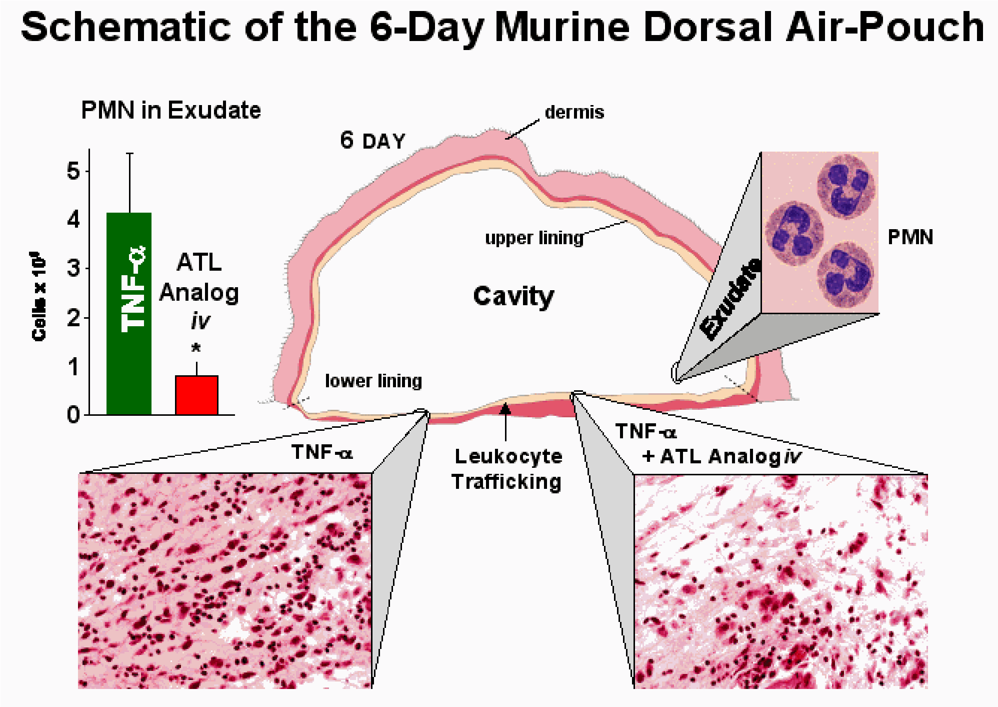

**Illustration 16.** The 6-day murine dorsal air-pouch is characterized by the presence of a nascent lining that encloses the air cavity. TNF-α induces leukocyte infiltration, predominantly PMN, which is inhibited by i.v. injection of ATL analog (see insets).

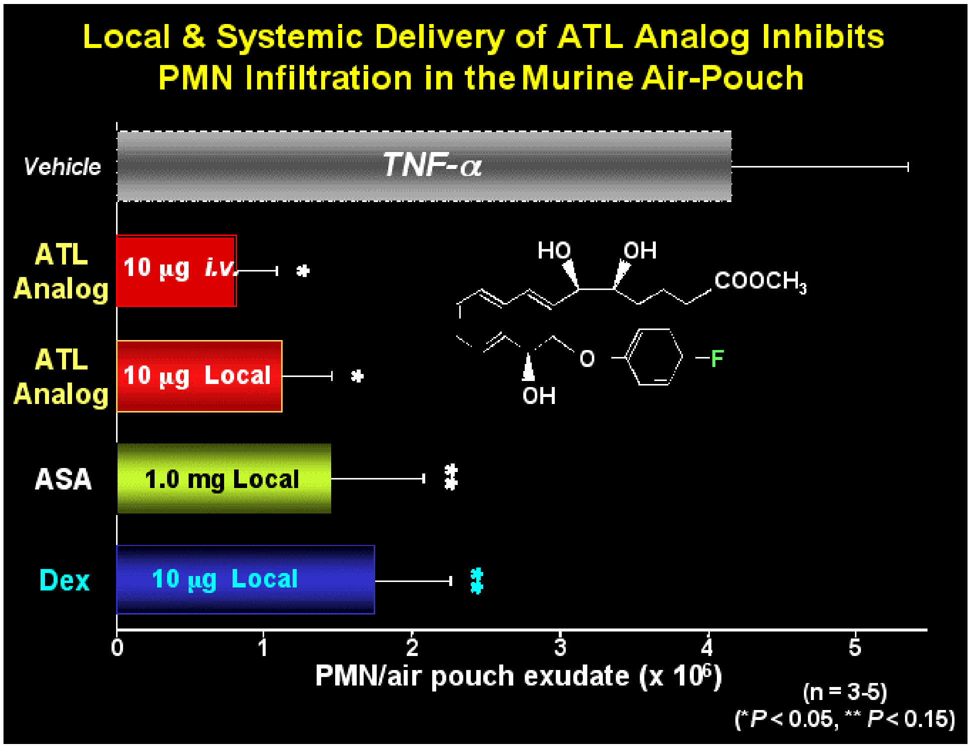

**Illustration 17.** Direct comparison of LX/ATL analogs with dexamethasone and ASA treatment. Note that the analog is >100 times more potent than ASA in preventing PMN entry[53].

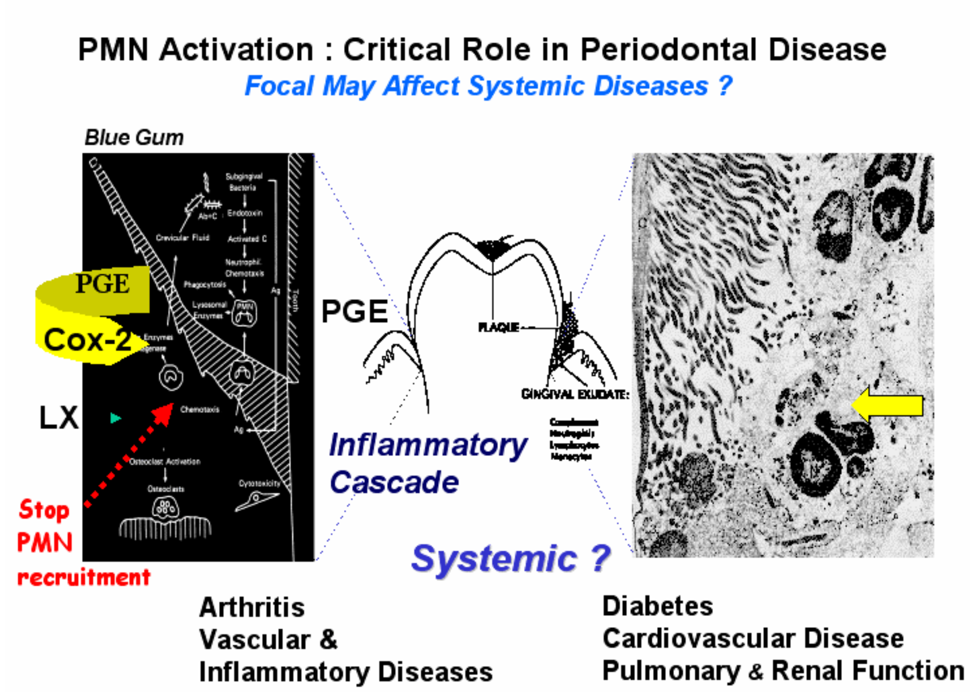

**Illustration 18.** Recruitment of PMN (right) followed by aberrant release of inflammatory mediators (left) contributes to the onset of periodontal disease and is associated with tissue destruction. The periodontal pathogen P. gingivalis-inflamed periodontal tissues display an increased $PGE_2$, a potent stimulator of bone loss, as well as upregulated COX-2 expression. LX stable analogs blocked P. gingivalis-elicited PMN infiltration and $PGE_2$ level when introduced into murine air-pouch cavity, suggesting that LX plays a novel protective role in periodontitis, limiting further PMN recruitment and PMN-mediated tissue injury.

### $LXA_4$ RECEPTORS

#### Molecular Cloning and Receptor Expression

The synthesis of the radiolabeled [11,12-$^3$H]-$LXA_4$[59] enabled the first direct characterization of specific $LXA_4$ binding sites present on PMN that are likely to mediate many of its selective actions on these cells[60]. Intact PMN demonstrate specific and reversible [11,12-$^3$H]-$LXA_4$ binding (Kd ~0.5 nM and $B_{max}$ ~1830 sites per PMN), which is modulated by guanosine stable analogs. These $LXA_4$ binding sites are inducible in promyelocytic lineage (HL-60) cells exposed to differentiating agents (e.g., retinoic acid, DMSO, and PMA) and confer $LXA_4$-stimulated phospholipase activation[61]. Together, these findings provided further evidence that $LXA_4$ interacts with specific membrane-associated receptors on human leukocytes that belong to the classical GPCR.

Based on our finding that functional ALXR are inducible in HL-60 cells, several putative receptor cDNAs that are also induced within this temporal frame, cloned earlier from myeloid lineages and designated orphans[62,63], were systematically examined for their ability to specifically bind and signal with $LXA_4$. Chinese hamster ovary (CHO) cells transfected with one of the orphans (denoted previously as pINF114, also known as FPRL1 and FPR2) display specific [$^3$H]-$LXA_4$ binding with high affinity (Kd = 1.7 nM) and demonstrated selectivity when compared to $LXB_4$, $LTB_4$, $LTD_4$, and $PGE_2$ (Illustration 13 and Table 4)[64]. These transfected CHO cells transmit signal with $LXA_4$, activating both GTPase and the release of arachidonic acid (C20:4) from membrane phospholipid, indicating that this cDNA encodes a functional receptor for $LXA_4$ in myeloid cells. The mouse ALXR cDNA was cloned from a spleen cDNA library and displays specific [$^3$H]-$LXA_4$ binding and $LXA_4$-initiated GTPase activity when transfected into CHO cells[55]. The human and mouse ALXR represented the first cloned LO-derived eicosanoid receptors. Both human[64] and mouse[55] ALXR cDNA contain an open reading frame of 1051 nucleotides, which encode a protein of 351 amino acids. Northern blot analysis demonstrated that ALXR mRNA is ~1.4 Kb in both human and mouse[55]. Chromosome mapping revealed that the gene encoding ALXR[64] is located on chromosome 19q[65], denoted as FPRH1 in this early report of the orphan receptor.

Northern blot analysis of multiple murine tissues demonstrated that ALXR mRNA is most abundant in PMN, spleen, and lung with lesser amounts in heart and liver[55]. In the absence of disease, the pattern is similar in human tissues. In humans, ALXR mRNA is also abundant in PMN followed by spleen, lung, placenta, and liver[55,64]. To date, ALXR is identified by function and direct actions, and cloned in both human and mouse PMN[55,64], human monocytes[66], and human enterocytes[50], as well as synovial fibroblasts[67]. In human PMN, results of subcellular fractionation experiments revealed that $[^3H]$-$LXA_4$ binding sites are associated with plasma membrane and endoplasmic reticulum (42.1%) and granule (34.5%) as well as nuclear-enriched fractions (23.3%), a distribution distinct from $[^3H]$-$LTB_4$ binding[60]. The finding that $LXA_4$ blocks both PAF and fMLP-stimulated eosinophil chemotaxis[57] suggests that functional ALXR is also present on eosinophils. In human enterocytes, ALXR is present in crypt and brush border colonic epithelial cells[50].

Retinoic acid, PMA and DMSO, which lead to granulocytic phenotypes in HL-60 cells, induce a approximate three- to fivefold increase in the expression of ALXR as monitored by specific $[^3H]$-$LXA_4$ binding[61] (see Table 7). Transcription of ALXR is dramatically upregulated by cytokines in human enterocytes, with lymphocyte-derived interleukin (IL)-13 and interferon-$\gamma$ being most potent, followed by IL-4 and IL-6. IL-1$\beta$ and LPS also showed moderate induction of ALXR mRNA[50]. In view of the cytokine regulation of ALXR, it is likely that the expression of these receptors will change dramatically in disease states, which in turn, might attenuate mucosal inflammatory and allergic responses.

### Structure-Function Relationships

Deduced amino acid sequence places ALXR within the GPCR superfamily characterized by seven putative transmembrane segments (TMS) with N-terminus on the extracellular side of the membrane and C-terminus on the intracellular side[68]. The overall homology between human and mouse ALXR is 76% in nucleotide sequence and 73% in deduced amino acid[55]. An especially high homology is evident for their second intracellular loop (100%) and between their sixth TMS (97%) followed by the second, third, and seventh TMS as well as the first extracellular loop (87 to 89%), suggesting essential roles for these regions in ligand recognition and G protein coupling. Molecular evolution analysis suggests that ALXR is only distantly related to prostanoid receptors and belongs to the cluster of chemoattractic peptide receptors exemplified by fMLP, C5a, and IL-8 receptors[69] and now known to also include BLT as well as the recently cloned cysteinyl-LT receptors (Illustration 11). BLT was obtained from human HL-60 cells[24] and mouse eosinophils[25] and found to share an overall ~30% homology with ALXR in deduced amino acid sequences. High homologous region (~46%) is present within the second transmembrane segments in both ALXR and BLT with the amino acid sequence LNLALAD. Prostanoids interact with their receptors via $COO^-$ interacting with an arginine residue within the seventh transmembrane segments[70]. Neither ALXR nor BLT share this Arg (in seventh transmembrane segments) requirement[24,64], yet both ligands contain COOH, which at physiological pH could present as a counter-anion. Together, these findings further provide evidence that the origin of receptors for LT and LX is distinct from that of receptors for prostanoids.

**TABLE 4**
**Competitive Binding of $^{3}$H-$LXA_4$ with Structure-Related Eicosanoids**

| | PMN | HL-60 | CHO-ALXR (human) | HEK-ALXR (human) | HUVEC |
|---|---|---|---|---|---|
| competition | $LXA_4$ (Kd= 0.5 nM)<br>$LXA_4$-methyl ester<br>15(R/S)-methyl-$LXA_4$<br>16-phenoxy-$LXA_4$<br>15-epi-$LXA_4$ | $LXA_4$ | $LXA_4$<br>(Kd= 1.7 nM) | $LXA_4$<br>$LXA_4$-methyl ester<br>15(R/S)-methyl-$LXA_4$<br>15-epi-16-(para-fluoro)-phenoxy-$LXA_4$ | $LXA_4$ (Kd = 11 nM)<br>$LTD_4$<br>SKF104353 ($CysLT_1$ antagonist) |
| partial competition | $LTC_4$ ($IC_{50}$= 62 nM)<br>$LTD_4$ ($IC_{50}$= 56 nM)<br>fMLP ($IC_{50}$ ~ 1,000 fold higher than $LXA_4$) | $LTC_4$ | $LTD_4$<br>(Ki= 80 nM)<br>fMLP | 15-deoxy-$LXA_4$ ($IC_{50}$ ~ 1,000 fold higher than $LXA_4$) | |
| no competition | $LTB_4$<br>$LXB_4$<br>6S-$LXA_4$<br>11-trans-$LXA_4$<br>SKF 104353 (also see Illustration #13) | $LTB_4$<br>ONO-4057<br>$LXB_4$<br>SKF 104353 | $LTB_4$<br>$LXB_4$<br>$PGE_2$ | $LTB_4$ | ONO-4057 ($LTB_4$ antagonist) |

### Ligand Binding Specificity: Peptide and Lipid Ligands

ALXR is stereoselective for its eicosanoid-based ligands. Table 4 summarizes the present knowledge of related lipid ligand affinity and specificity for ALXR. Intact human PMN and retinoic acid-differentiated HL-60 cells demonstrate specific and reversible [$^{3}$H]-$LXA_4$ binding with Kds ~0.5 and ~0.6 nM, respectively[60,61]. Several isomers of $LXA_4$ tested, namely 11-trans-$LXA_4$, 6S-$LXA_4$, and $LXB_4$, did not compete for these recognition sites, consistent with their functional responses in these systems. Results from Scatchard analyses indicate that [$^{3}$H]-$LXA_4$ binds PMN granule membrane-enriched fractions with comparable Kd (0.8 nM) but with a larger Bmax ($4.1 \times 10^{-11}$ M) than plasma membrane fractions (Kd = 0.7 nM, Bmax = $2.1 \times 10^{-11}$ M)[64]. Hence, it appears that additional receptors can be mobilized by granule fusion to the plasma membrane of PMN. [$^{3}$H]-$LXA_4$ specific binding is stereoselective, since $LTB_4$, $LXB_4$, 6S-$LXA_4$, 11-trans-$LXA_4$, or SKF104353 (a $CysLT_1$ antagonist) do not compete for [$^{3}$H]-$LXA_4$ in human PMN (Table 4 and Illustration 13).

### ALXR/FPRL Displays Multirecognition with Unrelated Peptide Ligands: A Possible Dual Function for the Receptor?

Human and mouse ALXR cDNA, each transfected into CHO cells, display specific binding with [$^{3}$H]-$LXA_4$, the human Kd 1.7 nM[64] and mouse Kd 1.5 nM[55], respectively. Human ALXR-transfected CHO cells were also tested for binding with other eicosanoids, including $LXB_4$, $LTD_4$, $LTB_4$, and $PGE_2$. Only $LTD_4$ shows competition with [$^{3}$H]-$LXA_4$ binding, giving a $K_i$ of 80 nM (Table 4)[64]. It is of interest to note that, although ALXR shares ~70% homology with FPR, ALXR binds [$^3$H]-fMLP with only low affinity (Kd ~5 μM) and proves to be selective for $LXA_4$ by 3 log orders of magnitude[71]. More recently, it was reported that certain peptides/proteins in the μM range can also interact with ALXR (as indicated above, is also known as FPRL-1) in *in vitro* model systems. These findings are summarized in Tables 5 and 6. The functional role(s) of these peptides in human biology, pertaining to their ability to activate FPRL-1, albeit at μM levels, remains of interest. The apparent $EC_{50}$ value for receptor activation (determined by mobilization of $[Ca^{2+}]_i$) by the best synthetic rogue peptide (e.g. MMK-1) of this synthetic series is approximately 2 nM[72], whereas $LXA_4$ and its analogs stimulate monocyte adherence via ALXR at concentrations less than 1 nM ($EC_{50}$ for analogs ~$8 \times 10^{-11}$ M, $EC_{50}$ for $LXA_4$ ~$8 \times 10^{-10}$ M)[66] or inhibit PMN transmigration and adhesion at $10^{-10}$ M[20]. These new findings suggest that small peptides as well as bioactive lipids can function as ligands for the same receptor, however with different affinity and/or distinct interaction sites within the receptor and separate intracellular signaling depending on the cell type and model system. Hence, it appears likely that the intracellular protein interactions following ligand-receptor binding are different for peptide vs. lipid ligands of this receptor because different conformations of the ligand-receptors are likely to be formed. Taken together, the finding that specific $LXA_4$-related structures (Illustration 13) and certain peptides interact with this receptor may reflect the need for mutlirecognition and receptor redundancies in the immune system in that the lipid ligands appear as endogenous ligands, while many of the peptides capable of activating ALXR/FPRL-1 *in vitro* are of exogenous origins, presumably microbial-derived peptides (Tables 5 and 6).

Among the related eicosanoid heteroligands tested in the HL-60 cell system, only $LTC_4$ at ~3-log molar excess competes for [$^3$H]-$LXA_4$ specific binding[58]. The cross competition of $LTC_4$ and $LTD_4$ observed with $LXA_4$ in several systems suggests that the "true" peptido-LT receptors may also be of this class of receptors. In several tissues and cell types other than leukocytes, results from pharmacological experiments indicate that $LXA_4$ acts via interacting with a subclass of peptido-LT receptors ($CysLT_1$) as a partial agonist to mediate its actions[60,73]. Along these lines, both $LTC_4$ and $LXA_4$, albeit at high concentrations (>1 μM), induce contractions of guinea pig lung parenchyma and release of thromboxane $A_2$ that is sensitive to $CysLT_1$-receptor antagonists[74], which is not likely to be a physiologic action of $LXA_4$. In certain cell types, $LXA_4$ (in the nanomolar range) blocks $LTD_4$ actions, and in this regard blocks specific [$^3$H]-$LTD_4$ binding to mesangial cells[73] and human umbilical vein endothelial cells (HUVEC)[55,61]. HUVEC specifically bind [$^3$H]-$LXA_4$ at a Kd of 11 nM, which can be inhibited by $LTD_4$ and SKF104353[61]. Therefore, it appears that $LXA_4$ interacts with at least two classes of cell surface receptors, one specific for $LXA_4$, which is present on leukocytes and enterocytes (ALXR) (see Illustration 14) the other shared by $LTD_4$, which is present on HUVEC and mesangial cells ($CysLT_1$) (Illustration 19 and 20). Along these lines, an inducible $CysLT_1$ was recently identified and cloned from HUVEC[75].

Recombinant $CysLT_1$ receptor gave stereospecific binding with both [$^3$H]-$LTD_4$ and a novel labeled mimetic of ATL ([$^3$H]-ATLa) that was displaced with $LTD_4$ and ATLa (~$IC_{50}$ 0.2 to 0.9 nM), and not with a biologically inactive ATL/LX isomer. In sharp contrast, $LTD_4$ was an ineffective competitive ligand for recombinant ALXR with [$^3$H]-ATLa. Endogenous murine $CysLT_1$ receptors also gave specific [$^3$H]-ATLa binding that was displaced with essentially equal affinity by $LTD_4$ or ATLa. Systemic ATLa proved to be a potent inhibitor (>50%) of $CysLT_1$-mediated vascular leakage in murine skin (200 μg/kg) in addition to its ability to block PMN recruitment to dorsal air-pouch (4 μg/kg). These results indicate that ATL and $LTD_4$ bind and compete with equal affinity at $CysLT_1$, providing a molecular basis for ATL serving as a local damper of both vascular $CysLT_1$ signals as well as ALXR-regulated PMN traffic[75].

### TABLE 5
## Comparison of Lipid vs. Peptide Ligands for Human ALXR in Phagocytic Cells

| *Ligand* | *PMN* | | *MONOCYTE* | |
|---|---|---|---|---|
| | *Ligand binding / Signaling* | *Concentration* | *Ligand binding / Signaling* | *Concentration* |
| **$LXA_4$ and ATLa** | - [$^3$H]-$LXA_4$ binding<br>- Inhibit transmigration and adhesion<br>- Inhibit chemotaxis | - Kd ~ 0.5 x$10^{-9}$ M<br>- ~$10^{-10}$ M<br>- peaked at $10^{-8}$ M | - Induces adherence | - $10^{-11}$ - $10^{-10}$ M |
| **MMK-1** | - Induces chemotaxis<br>- Displace [$^3$H]-$LXA_4$ binding | - ~3 x $10^{-9}$ M<br>- IC50 ~$10^{-11}$ M | | |
| **MHC peptide** | - Induces chemotaxis<br>- Displace [$^3$H]-$LXA_4$ binding | - ~3 x $10^{-9}$ M<br>- IC50 ~$10^{-11}$ M | | |
| **SAA** [112] | - [$^{125}$I]-SAA binding<br>- Induces chemotaxis<br>- $Ca^{2+}$ mobilization | - Kd ~ 45 x$10^{-9}$ M<br>- 0.8 – 4.0 x $10^{-6}$ M<br>- 0.8 – 4.0 x $10^{-6}$ M | - Induces chemotaxis<br>- $Ca^{2+}$ mobilization<br>- Increase CCR5 phosphorylation<br>- | - 0.8 – 4.0 x $10^{-6}$ M<br>- 0.8 – 4.0 x $10^{-6}$ M<br>- 1 – 20 µg/ml |
| **F-peptide (gp120 peptide)** [113] | - Induces chemotaxis<br>- $Ca^{2+}$ mobilization | - – 5.0 x $10^{-6}$ M<br>- 2.5 – 10.0 x $10^{-6}$ M | - Induces chemotaxis<br>- $Ca^{2+}$ mobilization<br>- Inhibits CCR5 and CXCR4<br>- binding and chemotaxis | - 1.0 – 5.0 x $10^{-6}$ M<br>- 1.0 – 10.0 x $10^{-6}$ M<br>- 1.0 – 5.0 x $10^{-5}$ M |
| **V3 peptide (gp120 peptide)** [114] | - Induces chemotaxis<br>- $Ca^{2+}$ mobilization | - 1 – 6 x $10^{-6}$ M<br>- 1 – 3 x $10^{-6}$ M | - Induces chemotaxis<br>- $Ca^{2+}$ mobilization<br>- Increase CCR5 phosphorylation | - 1 – 6 x $10^{-6}$ M<br>- 1 – 3 x $10^{-6}$ M |
| **T21/DP107 (gp41 peptide)** [115] | - Induces chemotaxis<br>- $Ca^{2+}$ mobilization | - $10^{-8}$ - $10^{-4}$ M<br>- $10^{-7}$ - $10^{-5}$ M | - Induces chemotaxis<br>- $Ca^{2+}$ mobilization | - $10^{-7}$ - $10^{-5}$ M |
| **N36 (gp41 peptide)** [116] | - Induces chemotaxis<br>- $Ca^{2+}$ mobilization | - $10^{-6}$ - $10^{-4}$ M<br>- 2.5 – 5 x $10^{-6}$ M | - Induces chemotaxis<br>- $Ca^{2+}$ mobilization | - $10^{-6}$ - $10^{-4}$ M<br>- $10^{-6}$ - $10^{-5}$ M |
| **WKYMVm** [117] | - Induces chemotaxis<br>- $Ca^{2+}$ mobilization<br>- Stimulate NADPH-oxidase activity<br>- Induce CD11b/CD18 mobilization | - $10^{-13}$ - $10^{-6}$ M<br>- $10^{-12}$ - $10^{-9}$ M<br>- EC50 ~ 2 x $10^{-9}$ M<br>- EC50 ~ 5 x $10^{-11}$ M | - Induces chemotaxis<br>- $Ca^{2+}$ mobilization | - $10^{-12}$ - $10^{-7}$ M<br>- $10^{-12}$ - $10^{-8}$ M |
| **LL-37** [118] | - Induces chemotaxis | - $10^{-6}$ - $10^{-5}$ M | - $Ca^{2+}$ mobilization<br>- Induces chemotaxis | - $10^{-6}$ - $10^{-5}$ M<br>- $10^{-6}$ - $10^{-5}$ M |
| **Amyloid $\beta_{42}$** [119] | | | - $Ca^{2+}$ mobilization<br>- Induces chemotaxis | - 5 x $10^{-6}$ M<br>- $10^{-6}$ - $10^{-4}$ M |
| **PrP106-126 (Neurotoxic prion peptide)** [120] | | | - Induces chemotaxis<br>- Production of pro-inflammatory cytokines | - $10^{-5}$ – $10^{-4}$ M<br>- 3 x $10^{-5}$ M |

**TABLE 6**
**Comparison of Lipid vs. Peptide Ligands for Recombinant Human ALXR**

| *Ligand* | *Cell type expressing recombinant human ALXR* | *LIGAND BINDING/SIGNALING* | *CONCENTRATION* |
|---|---|---|---|
| **$LXA_4$ and ATLa** | CHO<br>HEK293 | Induces chemotaxis<br>[$^3$H]-$LXA_4$ binding | $10^{-7}$ M<br>Kd ~ 1.5 nM |
| **MMK-1** | CHO<br>HEK293 | Induces chemotaxis<br>Displace [$^3$H]-$LXA_4$ binding | $10^{-9}$ M<br>IC50 ~$10^{-11}$ M |
| **MHC peptide** | HEK293 | Displace [$^3$H]-$LXA_4$ binding | IC50 ~$10^{-11}$ M |
| **SAA** [112] | HEK293 | Partially displace [$^3$H]-$LXA_4$ binding | IC50 ~$10^{-8}$ - $10^{-7}$ M |
| **F-peptide (gp120 peptide)** [113] | | | |
| **V3 peptide (gp120 peptide)** [114] | HEK293 | Induces chemotaxis<br>$Ca^{2+}$ mobilization | 1 – 3 x $10^{-6}$ M |
| **T21/DP107 (gp41 peptide)** [115] | HEK293 | Induces chemotaxis<br>$Ca^{2+}$ mobilization | $10^{-8}$ - $10^{-5}$ M<br>$10^{-8}$ - $10^{-6}$ M |
| **N36 (gp41 peptide)** [116] | HEK293 | Induces chemotaxis<br>$Ca^{2+}$ mobilization | $10^{-6}$ - $10^{-5}$ M<br>$10^{-6}$ - $10^{-5}$ M |
| **WKYMVm** [117] | HEK293<br>HL-60<br>(undifferentiated) | Induces chemotaxis<br>$Ca^{2+}$ mobilization | $10^{-13}$ - $10^{-6}$ M<br>EC50 ~7.5 x $10^{-11}$ M |
| **Amyloid $\beta_{42}$** [119] | HEK293 | Induces chemotaxis<br>$Ca^{2+}$ mobilization | $10^{-6}$ - $10^{-5}$ M<br>$10^{-6}$ - $10^{-5}$ M |
| **LL-37** [118] | HEK293 | Induces chemotaxis<br>$Ca^{2+}$ mobilization | $10^{-6}$ - $10^{-5}$ M<br>$10^{-6}$ - $10^{-5}$ M |

### Signal Transduction

The cytoplasmic signaling cascade of ALXR appears to be highly cell type-specific. For example, in human PMN $LXA_4$ stimulates rapid lipid remodeling (within seconds) with release of arachidonic acid that is evoked via PTX-sensitive G proteins[23] without formation of either LT or PG. Only a modest $Ca^{2+}$ mobilization was observed (Illustration 12). Also, $LXA_4$ was reported to block intracellular generation of $IP_3$[22] as well as $Ca^{2+}$ mobilization in response to other stimuli[56]. In human peripheral blood monocytes and cultured THP-1 cells, $LXA_4$ triggers intracellular $Ca^{2+}$ release and adherence to laminin[66,76]. Thus, different intracellular signaling pathways are present in PMN vs. monocytes despite identical receptor sequences (see Table 7 and Illustration 12). It is of interest that $Ca^{2+}$ is not the second messenger for LX actions in monocytes, since $LXA_4$-stimulated monocyte adherence to laminin is not dependent on a LX-stimulated increase in $[Ca^{2+}]_i$. The $EC_{50}$ value for $LXA_4$-stimulated increase in $[Ca^{2+}]_i$ is >100 nM in monocytes, which is more than 2 log orders of magnitude higher than that required for

**TABLE 7**
**Signal Transduction of Human and Mouse ALXR**

| *cell type* | *$LXA_4$ and ATLa-evoked signal transduction* | *kinase associated* | *Gene expression* | *up-regulated by* |
|---|---|---|---|---|
| **Human HL-60 (differentiated)** | • PLD activation (lipid remodeling) | protein kinase C (staurosporine sensitive) | | retinoic acid, DMSO, PMA |
| **Human PMN** | • PLD activation<br>• GTPase activity<br>• C20:4 release<br>• PIPP signal (↑PSDP accumulation) (with second signal)<br>• no increase of cAMP, proton efflux and very weak $[Ca^{2+}]_i$ | tyrosine kinase (genistein sensitive) | | |
| **Human monocyte** | • increase of $[Ca^{2+}]_i$ (PTX sensitive)<br>• no increase of cAMP and proton efflux | | | |
| **Human enterocyte** | • no proton efflux | | Reduce IL-8 mRNA level | IL-13, IL-4, interferon-γ |
| **Human synovial fibroblast** [67] | • PLD activation<br>• Inhibit NF-κB binding | | Stimulate TIMP transcription | |
| **CHO expressing human receptor** | • GTPase activity<br>• arachidonic acid release (PTX sensitive)<br>• no increase of cAMP and $[Ca^{2+}]_i$ | | | |
| **CHO expressing mouse receptor** | • GTPase activity | | | |

$LXA_4$-stimulated adherence ($EC_{50}$ <1 nM). In view of G-protein coupling events in monocytes, both $Ca^{2+}$ mobilization and adherence are PTX-sensitive. This indicates that receptor coupling in monocytes and PMN is similar to this point, although there could be different PTX-sensitive G-protein subtypes that couple to the intracellular domains of the receptors and diverge downstream in the signal transduction pathways leading to chemotaxis of monocytes and inhibition of PMN. The characteristics of ALXR in various cell types are briefly summarized in Table 6. Also, $LXA_4$ modulates MAP kinase activities on mesangial cells in a PTX-insensitive manner[77], suggesting the presence of additional novel ALXR subtypes and/or signaling pathways in these cells (Table 7).

In retinoic acid–differentiated HL-60 cells, $LXA_4$ stimulated PLD activation that is staurosporine sensitive, suggesting the involvement of PKC in signal transduction in these cells[61]. It was also demonstrated that $LXA_4$ blocks $LTB_4$ or fMLP-stimulated PMN transmigration or adhesion by regulation of β2 integrin–dependent PMN adhesion[71]. This modulatory action is partially reversed by prior exposure to genistein, a tyrosine kinase inhibitor[36]. In human renal mesangial cells, $LXA_4$ stimulates MAP kinase superfamily via two distinct receptors: one via a PTX-sensitive G protein, leading to p38 activation, and the other via a PTX-insensitive G protein, leading to ERK activation[78]. In human enterocytes (T84), ALXR activation by $LXA_4$ and LX analogs diminishes *Salmonella typhimurium*–induced IL-8 transcription[79]. The reduction of IL-8 mRNA level parallels decrements in IL-8 secretion, indicating that in these cells ALXR mechanism of action for blocking this chemokine is at the gene transcriptional level. In addition, $LXA_4$ induces tissue factor activity by increasing its mRNA level in EC304 cells (nonendothelial parenchymal cells) via a PTX-sensitive and PKC-dependent mechanism[80]. The ability of $LXA_4$ to induce tissue factor is an intriguing result. Its physiological role remains to be established in relation to LX generation and proximity to tissue factor releasing cells *in vivo*.

### Molecular Mechanism in Anti-Inflammation: Are Lipoxins and ATL Unique?

$LXA_4$, ATL, and their stable analogs activate ALXR, which then modulates PMN responses *in vitro*, such as chemotaxis, transmigration, adhesion, degranulation, cytokine release and functions, as well as inhibits PMN recruitment in several murine models. For example, ATL analog inhibits TNF-$\alpha$–initiated PMN infiltration in murine dorsal air-pouch[53] and $LTB_4$-induced PMN influx during dermal inflammation[55] as well as PMN-mediated second organ injury[81]. Recently, additional results from this laboratory indicated that, with PMN, ALXR interaction with LX and ATL analogs regulates a newly described polyisoprenyl phosphate (PIPP) signaling pathway[82] (Illustration 19). ALXR activation reverses $LTB_4$-initiated polyisoprenyl phosphate remodeling, leading to accumulation of presqualene diphosphate (PSDP), a potent negative intracellular signal in PMN which inhibits recombinant PLD and superoxide anion generation. When compared to other eicosanoids of COX, LO, and p450 products reported in the literature to display potential anti-inflammatory properties (see Table 9), LX and ATL stand apart both in mechanism and amount range for action. For example, $PGE_2$ reduced the antigen response[83] and inhibited macrophage phagocytosis[84], presumably via increasing intracellular cAMP levels, which in turn inhibits MAPK activation by stimulating PKA-dependent phosphorylation of Raf-1[85]. In contrast, $LXA_4$ does not give significant increase of cAMP levels in PMN[76]. In addition, cyclopenteneone PG such as 15-deoxy-$\Delta^{12,14}$-$PGJ_2$**,** in relatively high amounts, give anti-inflammatory action in adjuvant-induced arthritis in rats[86].

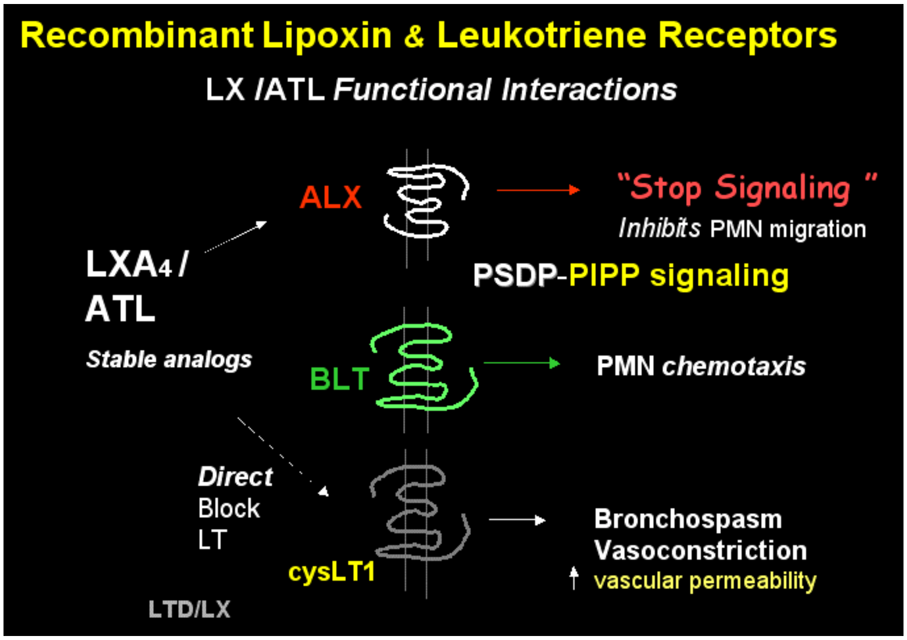

**Illustration 19.** $LXA_4$/ATL elicits bioactions with ALXR and a shared $CysLT_1$ subtype via direct ligand-receptor interaction and inhibits BLT bioactions via regulating intracellular PSDP-PIPP signaling.

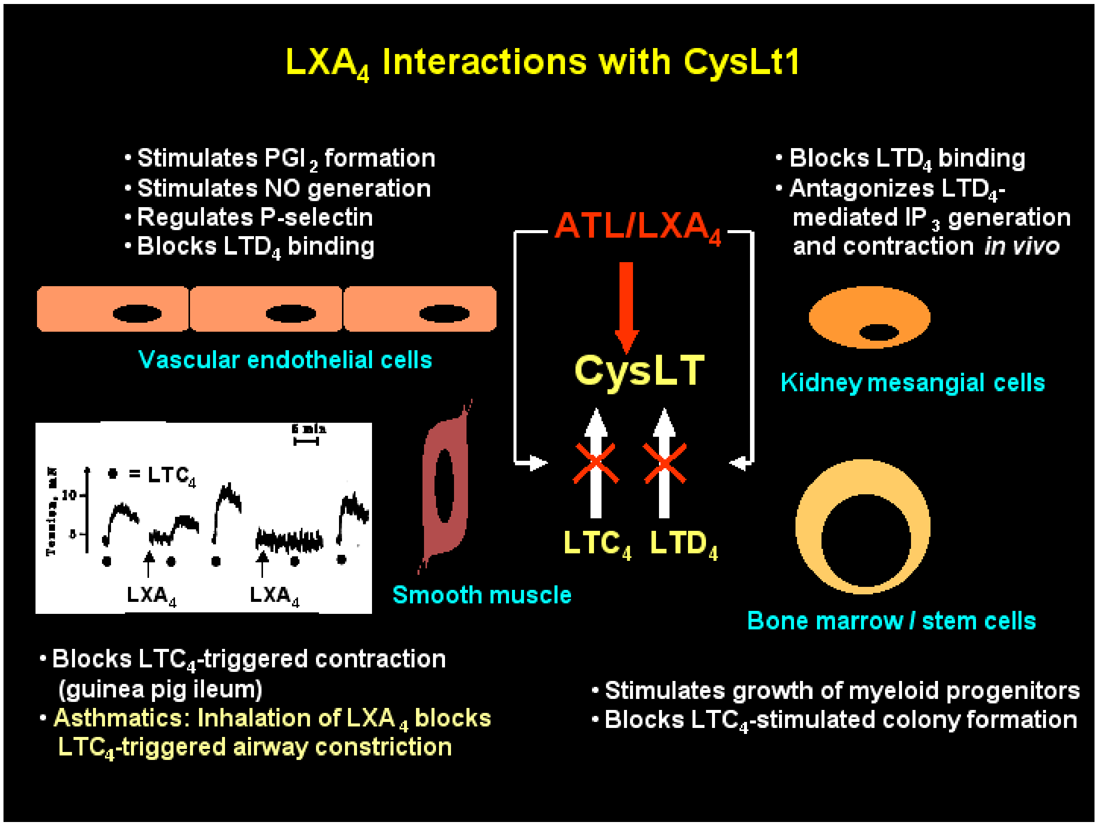

**Illustration 20.** Regulatory actions of $LXA_4$ in vascular endothelial cells, smooth muscle contraction and rat glomerular mesangial cells as well as in bone marrow via a subclass of peptido-LT receptors ($CysLT_1$).

**TABLE 8**
**$LXA_4$-Induced Signal Transduction via $CysLT_1$ or Other Receptors**

| *cell type* | *$LXA_4$ and ATLa-evoked signal transduction* | *kinase associated* | *Gene expression* |
|---|---|---|---|
| **Human endothelium** | • prostacyclin generation<br>• nitric oxide generation<br>• no increase of $[Ca^{2+}]_i$ and proton efflux | | |
| **Human renal mesangial cells** | | ERK (PTX-insensitive)<br>P38 (PTX-sensitive) | |
| **Parenchymal cells** | | protein kinase C | Increase tissue factor mRNA level (PTX and GF-109203X sensitive) |

**TABLE 9**
**Lipoxins and Other Potential Anti-Inflammatory Eicosanoids**

| **Lipid** | *Bioaction* | | ***Proposed mechanism*** |
|---|---|---|---|
| | Inhibition | Stimulation | |
| **COX product** | | | |
| **$PGE_2$** | • Macrophage phagocytosis (Rossi et al., 1998) [84]<br>• Acute allergic inflammation in hamster cheek pouches (Raud et al., 1988) [83] | • Potentiates $LTB_4$-induced vascular permeability change | ↑cAMP |
| **$PGI_2$** | • *LPS-induced TNF-$\alpha$ production (Guyton et al., 2001) [121]*<br>• *Human PMN chemotaxis (Nicolini et al., 1990) [122]* | • Inflammatory swelling and acetic acid writhing (Murata et al., 1997) [123]<br>• Enhances $LTB_4$-induced PMN infiltration in skin (Ekerdt et al., 1992) [124] | ↑cAMP |
| **$PGD_2$** | • granulocyte infiltration in colitis (Ajuebor et al., 2000) [125]<br>• Langerhans cell migration (Angeli et al., 2001) [126] | • eosinophil infiltration and airway hyperreactivity (Matsuoka et al., 2000) [127] | ↑cAMP (DP-dependent) |
| **Cyclopentenone prostaglandins** | | | |
| **15-deoxy-$\Delta^{12,14}$-$PGJ_2$** | • *adjuvant-induced arthritis in rats (Kawahito et al., 2000) [86]*<br>• *MCP-1 expression (Rovin et al., 2001) [128]*<br>• *LPS-induced TNF-$\alpha$ and NO production (Guyton et al., 2001) [121]* | | ↓NFkB and IkB kinase (GPCR-independent) (Rossi et al., 2000) [88] |
| **$PGA_2$** | • Viral replication (Santoro et al., 1988) [129] | | ↓NFkB and IkB kinase (GPCR-independent) |
| **LOX product** | | | |
| **$LXA_4$**<br>**$LXB_4$** | • PMN<br>• *allergic pleural eosinophil influx (Bandeira-Melo et al., 2000) [107] and duration of pleural exudation (Bandeira-Melo et al., 2000) [89]* | • Monocyte chemotaxis<br>• Uptake of apoptotic PMN by macrophages | ↑PSDP (in PMN) (ALXR-dependent in both PMN and monocytes) |
| **COX-LO product** | | | |
| **ATL** | • PMN<br>• *allergic pleural eosinophil influx (Bandeira-Melo et al., 2000) [107] and duration of pleural exudation (Bandeira-Melo et al., 2000) [89]* | • Monocyte chemoptaxis<br>• Uptake of apoptotic PMN by macrophages | ↑PSDP (in PMN) (ALXR-dependent in both PMN and monocytes) |
| **P450 product** | | | |
| **11,12-epoxy eicosatrienoic acid** | • PMN adhesion and VCAM-1 expression (Node et al., 1999) [130] | | ↓NFkB and IkB kinase<br>↑cAMP |

However, it appears that 15-deoxy-$\Delta^{12,14}$-$PGJ_2$ acts via mechanisms that are independent of cell surface GPCRs[87,88]. To date, $LXA_4$ and ATL are the only lipid mediators that possess anti-inflammatory and proresolution properties acting in the nanomolar range, since they regulate leukocyte trafficking and contribute to the early resolution of allergic pleural edema[89].

### SUMMARY

LX are the trihydroxytetraene-containing class of eicosanoids primarily generated by cell-cell interactions via transcellular biosynthesis that serve as local endogenous anti-inflammatory mediators. These “stop signals” in inflammation and other related processes may be involved in switching the cellular response from additional PMN recruitment toward monocytes (in a nonphlogistic fashion) that could lead to resolution of the inflammatory response and/or promotion of repair and healing. ASA impinges on this homeostatic system and evokes the endogenous biosynthesis of the carbon-15 epimers of LX, namely ATL, that mimic the bioactions of native LX in several biological systems (see Tables) and can thus modulate in part the beneficial actions of ASA in humans.

$LXA_4$ elicits biological actions via at least two main classes of receptor systems known to date: (1) ALXR on leukocytes and enterocytes and (2) a shared $CysLT_1$ subtype on endothelial and mesangial cells. ALXR belongs to the classical GPCR and was identified in mammalian tissues and characterized using direct evidence obtained with specific [$^3$H]-$LXA_4$ binding and activation of functional responses with $LXA_4$. ALXR is the first cloned LO-derived eicosanoid receptor; ALXR and BLT are more akin to chemokine receptors in their deduced amino acid sequences than the currently known prostanoid receptors. The cytoplasmic signaling pathways and bioactions of ALXR are cell type-specific. In human PMN, $LXA_4$ stimulates rapid lipid remodeling with release of arachidonic acid in a PTX-sensitive fashion, but does not trigger significant increases in intracellular $Ca^{2+}$. $LXA_4$ inhibits PMN adhesion, chemotaxis, and transmigration as well as degranulation and was implicated as an endogenous “stop signal” acting on PMN. In human monocytes, LX/ATL are potent chemoattractants and, in THP-1 cells and monocytes, LX/ATL initiate intracellular $Ca^{2+}$ release via ALXR, but neither $Ca^{2+}$ nor cAMP proved to be the required second messengers of LX actions in these cell types, indicating different intracellular signaling pathways despite identical receptor cDNA sequences. $LXA_4$ stimulates chemotaxis and adherence in monocytes but no apparent “proinflammatory” responses of these cells *in vitro or in vivo*, findings that may relate to the recruitment of monocytes to sites of wound healing and clearance. Indeed, LX and ATL stimulate the uptake of apoptotic PMN by macrophages in a nonphlogistic fashion[90].

The activation of a LX biosynthetic circuit *in vivo* requires upregulation of key enzymes by cytokines such as IL-4 and IL-13 (Illustration 5) that also control the expression of the receptor ALXR[50]. Moreover, both the temporal and spacial components in LX formation and actions are important determinants in their bioimpact during an acute inflammatory reaction[91]. LX and ATL appear to be the first recognized members of a new mediator class; namely, endogenous mediators of anti-inflammation. $PGE_2$ may display anti-inflammation in certain settings[92], but in most it enhances inflammation *in vivo*[54]. This is likely the result of numerous receptor isoforms and differential coupled mechanisms for $PGE_2$ and its diverse role in human physiology. Hence, the ability of $PGE_2$ to stimulate expression of 15-LO and set in place LX biosynthesis suggests that inhibition of PG can delay the onset of LX biosynthesis and prolong resolution[91].

The relationship between LX generation and current NSAID therapies is more intertwined than currently appreciated[93] in that ASA inhibits COX-1 and converts COX-2 into an ASA-triggered lipid mediator-generating system that produces an array of novel compounds from polyunsaturated fatty acids including arachidonic acid and eicosapentaenoic acid (EPA for example), some of which display potent anti-inflammatory or anti-PMN recruitment activity[94] as well as impinge on the role of these compounds in resolution. Results with ATL and LX analogues reviewed here show highly potent stereoselective actions in the sub- to nanomolar range sustaining LX and ATL actions in several *in vivo* models, indicating that these pathways (Illustrations 5, 6, 7) are likely to be important *in vivo* in human host defense. They join the many mediators that govern this process *in vivo* such as select cytokines (IL-10, IL-4, IL-13), proteins of interest in resolution[9]. In this regard, LX and ATL receptor activation not only inhibits proinflammatory events such as IL-6 gene expression[67] but stimulates IL-4 generation *in vivo*[95] and stimulates the phagocytosis of apoptotic PMN by macrophages[90]. The integrated response of the host is essential to health and disease; thus, it is important to achieve a more complete understanding of the molecular and cellular events governing the formation and actions of endogenous mediators of resolution that appear to control the duration of inflammation. Hence, it is not surprising that others have recently found a protective action for COX-2 in cardiovascular disease[96]. Establishing useful experimental systems will also take a multidisciplinary approach and require a shift in our current thinking about inflammation and the role of lipid mediators in its natural resolution. In addition, it appears that LX also display organ-specific actions in addition to host defense and immune roles such as the eye, kidney, lung, oral and GI tract and within bone marrow progenitors, possibly involving stem cells (Tables 1 and 3).

In this context, the words of Francis Bacon (1561–1620) tell of this dilemma: *"Contemplation's of nature and of bodies in their simple form break up and distract the understanding, while contemplation's of nature and bodies in their composition and configuration overpower and dissolve the understanding... for that school is so busy with particles that it hardly attends to the structure, while the others are so lost in admiration of the structure that they do not penetrate to the simplicity of nature."*

In agreement with *in vitro* results, ALXR agonists, namely $LXA_4$ and 15-epi-$LXA_4$ as well as their stable analogs, are topically active in inhibiting PMN infiltration as well as vascular permeability in murine skin inflammation. The development of these relatively few synthetic stable analogs has already provided valuable tools to evaluate the biological roles, significance, and pharmacological actions of ALXR as well as provided a novel means to selective therapies for inflammatory diseases. Along these lines, we recently reported that ASA and other NSAIDs together with dietary omega-3 polyunsaturated fatty acid[94] (ω-3 PUFA) supplementation stimulate the generation of a novel array of bioactive compounds, i.e., 5,12,18*R*-tri HEPE derived from the interactions of ω-3 with COX-2 and ASA (Illustration 21). This uncovers entirely new biochemical pathways such as the 18R series from eicosapentanoic acid and might provide a basis to explain the beneficial actions of fish oil-based treatment reported for many human diseases[97]. These novel ATL mediators from cell-cell interactions with ω-3 conversion by COX-2 give novel ligands and biotemplates to further explore the receptors and critical pathways in endogenous anti-inflammation and expediting resolution. They also underscore the important role of the vascular endothelium in generating biogenetic intermediates and as a vast metabolic organ in and of itself[94] (Illustrations 6, 7, and 21). Together with the LX and 15-epi-LX, the identification of these novel endogenous anti-inflammatory lipid mediators[94] gives us new avenues of approach in considering therapeutics for inflammation, cardiovascular diseases, and cancer (http://letheon.bwh.harvard.edu/research/overview/cet+ri.phtml).

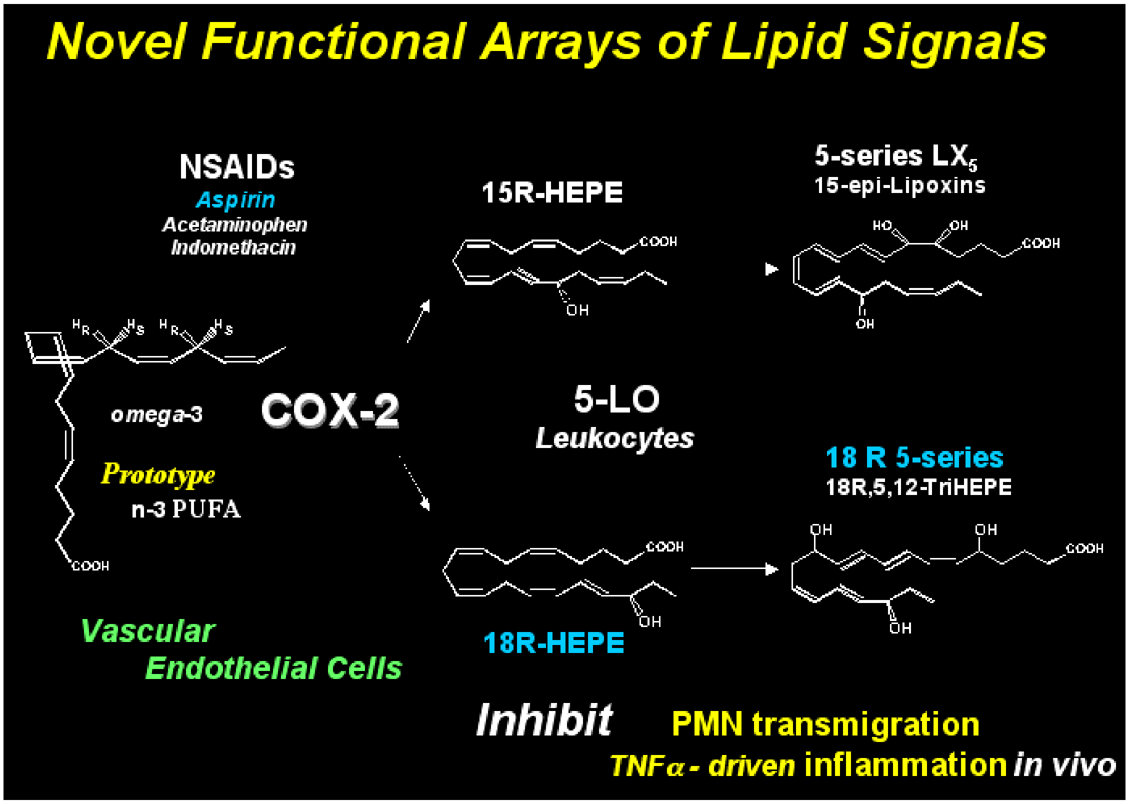

**Illustration 21.** Interactions of omega–3 polyunsaturated fatty acids (ω-3 PUFA) with ASA-acetylated COX-2, which generate novel arrays of bioactive compounds, such as 5,12,18R-tri HEPE or 15R-LX/ATL, each of which inhibits PMN transmigration in vitro and inflammation in vivo. A prototypic oxygenation with EPA is depicted as an ω-3-containing fatty acid[94].

### ACKNOWLEDGMENTS

The author's laboratory is supported in part by National Institutes of Health grant nos. GM38765, DK50305, and P01-DE13499. The authors thank Mary Halm Small for expert assistance in manuscript preparation.
